# Gamma-aminobutyric acid type A receptor alpha 4 coordinates autophagy, inflammation, and immunometabolism to promote innate immune activation

**DOI:** 10.1080/27694127.2023.2181915

**Published:** 2023-03-01

**Authors:** Jin Kyung Kim, Prashanta Silwal, Young Jae Kim, Sang Min Jeon, In Soo Kim, June-Young Lee, Jun Young Heo, Sang-Hee Lee, Jin-Woo Bae, Jin-Man Kim, Jin Bong Park, Eun-Kyeong Jo

**Affiliations:** a Department of Microbiology, Keimyung University School of Medicine, Daegu, Korea; b Department of Microbiology, Chungnam National University School of Medicine, Daejeon, Korea; c Infection Control Convergence Research Center, Chungnam National University School of Medicine, Daejeon, Korea; d Department of Medical Science, Chungnam National University School of Medicine, Daejeon, Korea; e Department of Biology and Department of Biomedical and Pharmaceutical Sciences and Department of Life and Nanopharmaceutical Sciences, Kyung Hee University, Seoul, Korea; f Department of Biochemistry, Chungnam National University School of Medicine, Daejeon, Korea; g Center for Research Equipment, Korea Basic Science Institute, Cheongju, Korea; h Department of Pathology, Chungnam National University School of Medicine, Daejeon Korea; i Department of Physiology, Chungnam National University School of Medicine, Daejeon, Korea

**Keywords:** GABRA4, Mycobacteria, infection, inflammation, AMPK, macrophages, mitochondrial oxidative phosphorylation, immunometabolism, sepsis

## Abstract

Gamma-aminobutyric acid type A receptor (GABA_A_R), the ionotropic receptor of GABA, is expressed in macrophages and in the nervous system; however, its role in innate immunity is unknown. Herein, we identified myeloid GABA_A_R subunit α4 (*Gabra4*) as a critical regulator of autophagy and a promoter of host innate defense during infection and inflammation. Myeloid *Gabra4* deficiency led to defective mycobacterial clearance during infection and increased susceptibility to septic shock. *Gabra4* deletion exaggerated inflammatory responses and suppressed the activation of autophagy in macrophages upon infectious and inflammatory stimuli. Mechanistically, *Gabra4*-mediated signaling led to upregulation of autophagy in macrophages via intracellular calcium release and AMP-activated protein kinase (AMPK) signaling activation, which was required for linking autophagy and antimicrobial responses. Additionally, *Gabra4* was required to generate mitochondrial reactive oxygen species, thereby triggering autophagy and antimicrobial responses to mycobacteria. Metabolomics analysis showed that *Gabra4* was critical for glucose metabolism and aerobic glycolysis in macrophages. Our findings demonstrate that myeloid *Gabra4* coordinates autophagy, inflammation, and immunometabolism to promote innate host defense against pathogenic and dangerous stimuli.

**Abbreviation** AM: Alveolar macrophage; AMPK: AMP-activated protein kinase; ASC: Apoptosis-associated speck-like protein containing a CARD; ATP: Adenosine 5’-triphosphate; BAL: Bronchoalveolar lavage; BCG: *Mycobacterium bovis* Bacillus Calmette–Guérin; BMDM: Bone marrow-derived macrophage; CCL: CC motif chemokine ligand; CFU: Colony forming unit; CKO: Conditional knock out; CXCL: C-X-C motif ligand; Dpi: Days post-infection; ECAR: Extracellular acidification rate; EGFP: Enhanced green ﬂuorescent protein; FOXO3: Forkhead box O3; GABA: Gamma-aminobutyric acid; GABA_A_R: GABA type A receptor; Gabarap: GABA type A receptor-associated protein; Gabarapl1: GABA type A receptor-associated protein like 1; GABRA4: Gamma-aminobutyric acid type A receptor subunit alpha4; HIF-1α: Hypoxia-inducible factor-1 alpha; IL: Interleukin; i.n.: Intranasal; i.p.: Intraperitoneal; LDHA: Lactate dehydrogenase A; LPS: Lipopolysaccharide; Mabc: *Mycobacteroides abscessus* subsp. *abscessus*; MOI: Multiplicities of infection; Mtb: *Mycobacterium tuberculosis*; mtROS: Mitochondrial reactive oxygen species; OCR: Oxygen consumption rate; OXPHOS: Oxidative phosphorylation; PM: Peritoneal macrophage; TNF: Tumor necrosis factor; WT: Wild type

## Introduction

Gamma-aminobutyric acid (GABA), a primary inhibitory neurotransmitter, contributes to neurogenesis, network formation, and chloride homeostasis in the central nervous system [1]. Beyond this, GABA functions as an intercellular signaling molecule in the immune system, particularly in macrophages [2-5]. GABA acts via GABA A receptors (GABA_A_Rs) and GABA_B_Rs, which are mainly distributed in the central nervous system, to regulate neuronal excitability in the central nervous system [6,7]. An extraneuronal function of GABA_A_Rs reportedly depends on the distribution of receptor subtypes in multiple cell types and tissues [8-13]. GABA_A_Rs are heteropentameric GABA-gated ion channels made of at least 19 subunits [6,14,15]. Among the receptor subtypes that comprise GABA_A_Rs, α1-3βγ are typical components of synaptic GABA_A_Rs, whereas α4βδ, α5βγ, and αβ are extrasynaptic isoforms that underlie tonic inhibition [8,14,16]. Despite this, it is unknown which receptor subunit of GABA_A_R regulates host defense in macrophages, the principal innate immune cells. Determining the role of GABA_A_Rs in the peripheral immune system would advance efforts to develop novel therapeutic approaches for infection.

Balanced regulation between innate immunity and inflammation is critical for host resistance against pathogens and stress stimuli [17,18]. Autophagy, a cell-autonomous host defensive pathway, is required to maintain intracellular homeostasis and orchestrates innate immunity, inflammation, and immunometabolic signaling [19-21]. The coordinated action of autophagy and innate immune signaling protects against intracellular microbes by supporting antimicrobial defense and controlling harmful excessive inflammation [19,22,23]. If autophagy regulation fails, exaggerated inflammatory responses and dysregulated inflammasome complex activation render the host vulnerable to infection [24-26]. Furthermore, autophagy interconnects with mitochondrial metabolism, through which immune cell function is regulated during infection [19,27]. Two key metabolic regulators, AMP-activated protein kinase (AMPK) and mammalian target of rapamycin (mTOR) kinase, are located in lysosomes, respond to infectious stimuli, and are critical mediators of the interplay between autophagy and immunometabolism, influencing host defense against intracellular bacterial infection [27]. However, it is unknown whether and how autophagy, inflammation, and immunometabolism are coordinated in the context of host defense and immune responses during infection and inflammation.

GABA_A_R subunit α4 (GABRA4) is a major GABA_A_R subunit expressed in bone marrow-derived macrophages (BMDMs) of mice [3]. Here, we identify GABRA4 as a pivotal orchestrator of autophagy, inflammatory responses, and immunometabolism in macrophages and a promoter of innate immunity. Myeloid *Gabra4* deficiency led to defective bacterial clearance during mycobacterial infection and increased lethality to lipopolysaccharide (LPS)-induced septic shock. *Gabra4* expression in myeloid cells is critical for activating autophagy and controlling excessive inflammatory responses during infection and inflammation. Mechanistically, GABRA4 triggers the expression of *Map1lc3a* and *forkhead Box O3* (*Foxo3*) in macrophages in response to mycobacterial infection. In addition, autophagy induced by *Mycobacterium tuberculosis* (Mtb) or LPS is dependent on intracellular Ca^2+^ release and AMPK activation in macrophages. Importantly, GABRA4-mediated AMPK signaling was required to orchestrate autophagy, inflammation, and mitochondrial oxidative phosphorylation (OXPHOS) during Mtb infection. Moreover, GABRA4 is required to maintain mitochondrial respiration to generate mitochondrial reactive oxygen species (mtROS), which triggers autophagy and antimicrobial responses in the early phase of infection. GABRA4 is also required for the optimal glycolytic activity, which might be related to host defensive responses in macrophages. These findings indicate that GABRA4 is a critical coordinator of autophagy, inflammation, and immunometabolism, and a gatekeeper of host defense against pathogenic and dangerous stimuli.

## Results

### GABRA4 is necessary for host defense against mycobacterial infection *in vivo* and in macrophages

**Figure 1.**
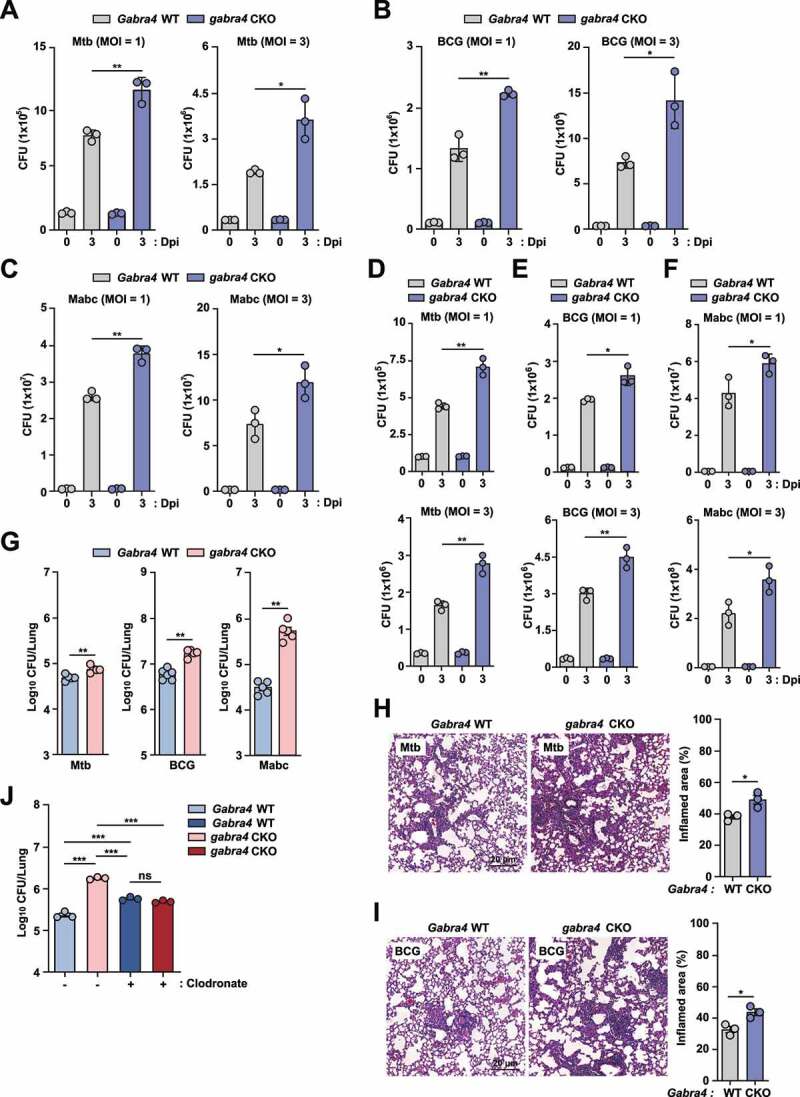
GABRA4 is essential for macrophage antimicrobial responses against mycobacterial infection. (**A-F**) Survival of bacteria after Mtb (A, D), BCG (B, E), and Mabc (C, F) infection (MOI 1 and 3) in *Gabra4* WT and *gabra4* CKO BMDMs (A–C) and PMs (D–F). (**G**) Bacterial load in lungs of *Gabra4* WT and *gabra4* CKO mice infected with Mtb (5 × 10^4^ CFU) at 10 dpi, BCG (1 × 10^7^ CFU) and Mabc (1 × 10^7^ CFU) at 7 dpi. (**H, I**) H&E staining of *Gabra4* WT and *gabra4* CKO mice infected with Mtb (H) and BCG (I) for 21 and 14 days, respectively showing the lung histopathology. Bar graphs show the quantification of results. Scale bar, 20 μm. (**J**) Bacterial load in control (liposome) and clodronate-treated *Gabra4* WT and *gabra4* CKO mice lungs after infection with Mabc (1 × 10^7^ CFU) at 7 dpi. Means ± SEM are shown (A–J). The Mann-Whitney U test was used to assess significance. Representatives of three independent experiments are shown. *p < 0.05, **p < 0.01, ***p < 0.001. ns, not significant; Dpi, days post-infection.

Given that GABRA4 is the major subunit of GABA_A_R expressed in primary BMDMs from mice [3], we investigated the innate immune function of GABRA4 in macrophages. To examine this, we generated *gabra4* conditional knockout (CKO) mice by crossing the *Gabra4^fl^°^x^*^/^*^fl^°^x^* and myelomonocytic cell-specific lysozyme M (LysM)-Cre mice. Genotyping of each mouse was performed using the tail genomic DNA to confirm the presence of flox allele in littermate control (*Gabra4* wild type [WT]) and the mutant allele in *Gabra4* (*gabra4* CKO)-deficient mice (Fig. S1A). We first compared the antimycobacterial activities of *Gabra4* WT and *gabra4* CKO BMDMs. *Gabra4* WT and *gabra4* CKO BMDMs were infected with *Mycobacterium tuberculosis* (Mtb), *M. bovis* Bacillus Calmette–Guérin (BCG), and *Mycobacteroides abscessus* subsp. *abscessus* (Mabc) and subjected to intracellular colony-forming unit (CFU) assays. Notably, *gabra4* CKO BMDMs had a significant increase in intracellular bacterial growth at multiplicities of infection (MOI) of 1 or 3, compared to *Gabra4* WT BMDMs (Fig. 1A-C) at 3 days post-infection (dpi). In peritoneal macrophages (PMs), *Gabra4* expression was lower than that of *Gabra6* (Fig. S1B). Similar to BMDMs, intracellular mycobacterial survival was increased in *gabra4* CKO PMs compared to *Gabra4* WT PMs (Fig. 1D–F). However, there was no significant difference in the phagocytic abilities of BMDMs or PMs from *Gabra4* WT and *gabra4* CKO mice (Fig. S1C and S1D).

Next, we investigated the role of GABRA4 in antimicrobial host defense *in vivo*. When *Gabra4* WT and *gabra4* CKO mice were intranasally infected with Mtb, BCG, and Mabc, the bacterial loads were significantly higher in *gabra4* CKO mice lung as compared to *Gabra4* WT mice lung (Fig. 1G, Mtb at 10 dpi; BCG and Mabc at 7 dpi). We also noted that Mtb or BCG infection led to significantly higher number of granulomatous lesions in the lung tissues from *gabra4* CKO mice (Fig. 1H for Mtb; Fig. 1I for BCG) than in *Gabra4* WT mice. Given that *in vivo* bacterial load is higher in the *Gabra4*-deficient lung, we questioned whether alveolar macrophages (AMs) play a role in GABRA4-mediated host defense during infection. To examine this, we examined the expression profiles of GABA_A_R subunits in AMs prepared from the bronchoalveolar lavage (BAL) fluid of *Gabra4* WT mice. Although *Gabra4* expression was lower than other subunits (such as *Gabra5*) in AMs (Fig. S1E), intracellular Mtb growth was significantly increased in AMs from *gabra4* CKO mice than those from *Gabra4* WT mice (Fig. S1F). These findings suggest that GABRA4 is crucial for the antimicrobial responses of a variety of macrophage subtypes.

We next examined whether macrophages are responsible for the differences in antimicrobial host defense between *Gabra4* WT and *gabra4* CKO mice. By using clodronate-liposomes, macrophages were depleted in *Gabra4* WT and *gabra4* CKO mice prior to infection. As shown in Fig. 1J, macrophage depletion attenuated the differences in antimicrobial activity between *Gabra4* WT and *gabra4* CKO mice. Interestingly, macrophage depletion of *gabra4* CKO mice significantly suppressed the pulmonary Mabc load to a level similar to *Gabra4* WT mice (Fig. 1J). Collectively, these results indicate that myeloid GABRA4 enhances antimicrobial activity during mycobacterial infection. In addition, macrophages are implicated in GABRA4-mediated host defense during mycobacterial infection.

### GABRA4 signaling attenuates pathologic inflammation against mycobacterial infection, *in vivo* and in macrophages

**Figure 2.**
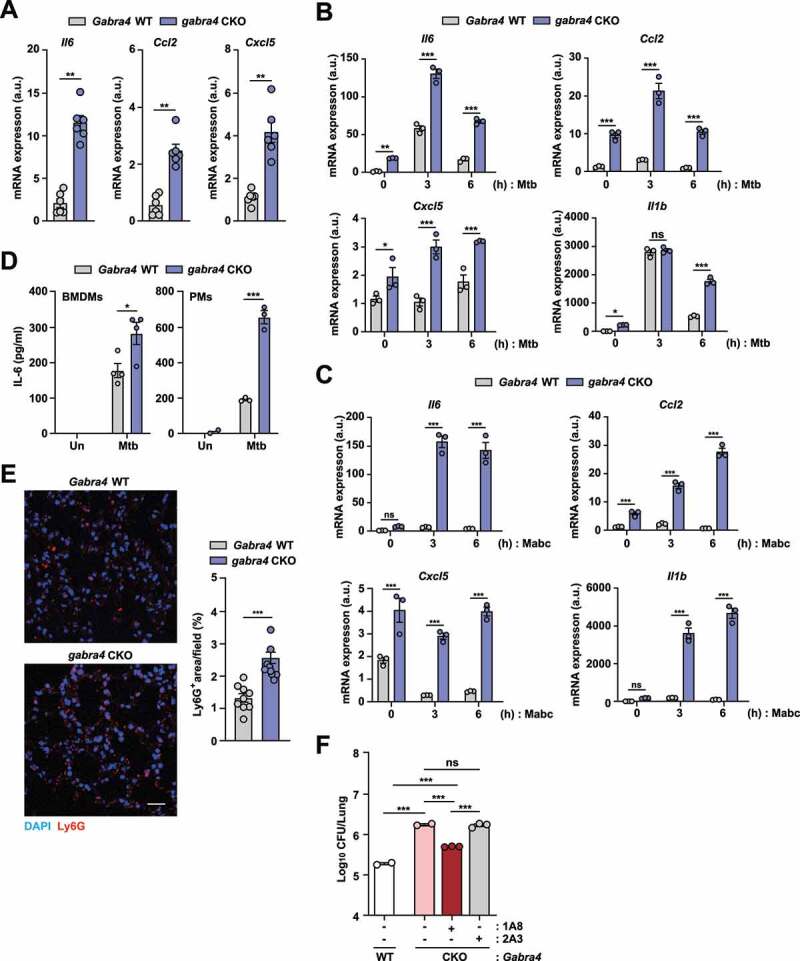
GABRA4 is essential to control pathologic inflammation during mycobacterial infection. (**A**) qRT-PCR analysis of *Il6, Ccl2*, and *Cxcl5* mRNA expression in the lungs of Mtb-infected *Gabra4* WT and *gabra4* CKO mice. (**B, C**) qRT-PCR analysis of *Il6, Ccl2, Cxcl5*, and *Il1b* mRNA levels in Mtb- (**B**, MOI 1) and Mabc- (**C**, MOI 1) infected *Gabra4* WT and *gabra4* CKO PMs. (**D**) IL-6 level in the supernatant after 18 h of Mtb (MOI 1) infection in *Gabra4* WT and *gabra4* CKO BMDMs or PMs. (**E**) Ly6G-stained neutrophils in lung tissues from *Gabra4* WT and *gabra4* CKO mice infected with Mtb (5 × 10^4^ CFU) at 21 dpi. Scale bar, 25 μm. (**F**) *Gabra4* WT or *gabra4* CKO mice were infected with Mabc (1 × 10^7^ CFU) at 7 dpi and treated with the neutrophil-specific anti-Ly6G (clone 1A8) or isotype control (clone 2A3) antibody. Bacterial burden in lung tissues. Means ± SEM (A, D, and F) or ± SD (B, C, and E right). Mann-Whitney U test (A), two-way ANOVA (B, C), unpaired *t*-test (D, E right), or one-way ANOVA (F) was used to assess significance. The data shown are representative of three independent experiments. *p < 0.05, **p < 0.01, ***p < 0.001. ns, not significant; Un, uninfected.

Because dysregulated inflammatory responses are associated with immune pathogenesis and tissue destruction in tuberculosis [28,29], we then tested to see whether *Gabra4* deficiency is linked to altered regulation of inflammatory responses. qRT-PCR showed that the mRNA expressions of proinflammatory cytokines and chemokines—i.e., interleukin (*Il*)*6,* CC motif chemokine ligand (*Ccl*)*2*, and C-X-C motif ligand (*Cxcl*)*5*—were significantly higher in the lung tissue of Mtb-infected *gabra4* CKO mice compared to *Gabra4* WT mice (Fig. 2A). Next, we infected PMs from *Gabra4* WT and *gabra4* CKO mice with Mtb or Mabc, and found that the levels of proinflammatory cytokines and chemokines mRNA—i.e., *Il6, Ccl2, Cxcl5*, and *Il1b*—were significantly higher in *gabra4* CKO PMs at 3 and 6 h of infection (Fig. 2B for Mtb; Fig. 2C for Mabc) as compared to *Gabra4* WT PMs. The protein level of IL-6 was significantly increased in *gabra4* CKO macrophages compared to *Gabra4* WT macrophages (Fig. 2D), suggesting that myeloid GABRA4 contributes to the amelioration of excessive inflammatory responses during mycobacterial infections.

To confirm a role for GABRA4 in the resolution of inflammation, we compared the expression of anti-inflammatory mediators, such as *Il10* and *Arg1*, in macrophages and lungs between *Gabra4* WT and *gabra4* CKO mice. The *Il10* mRNA level at 6 and 18 h of Mtb infection was not significantly different between *Gabra4* WT and *gabra4* CKO BMDMs (Fig. S2A). Although *Arg1* expression was markedly lower at 0–6 h after Mtb infection, it was increased at 18 h in *gabra4* CKO BMDMs compared to *Gabra4* WT BMDMs (Fig. S2B). Moreover, *Arg1*, but not *Il10*, was decreased in the lung tissues from *gabra4* CKO mice compared to those from *Gabra4* WT mice (Fig. S2C). Therefore, the upregulated inflammatory signaling *in vivo* induced by *Gabra4* deficiency is not associated with the downregulation of anti-inflammatory responses.

We further examined how GABRA4 signaling contributes to the regulation of pulmonary inflammation during infection. Interestingly, AMs from *gabra4* CKO mice showed decreased proinflammatory cytokine generation compared to those from *Gabra4* WT mice (Fig. S2D and S2E), suggesting that AMs are not the major cell types responsible for the GABRA4-mediated control of proinflammatory responses *in vivo*. Excessive infiltration of neutrophils induces pathological inflammation and increases the bacterial burden in the lung during mycobacterial infection [30-32]. So, we checked whether neutrophil infiltration differed between the lungs from *Gabra4* WT and *gabra4* CKO mice. As shown in Fig. 2E, neutrophil infiltration in the lung was markedly higher in *gabra4* CKO mice compared to *Gabra4* WT mice after Mtb infection. Notably, treatment of *gabra4* CKO mice with anti-neutrophil antibodies (1A8) significantly attenuated Mabc growth in the lung compared to the control (isotype control antibody, 2A3; Fig. 2F). Therefore, during mycobacterial infection, increased neutrophil infiltration but not AMs, may contribute to pathological inflammatory responses in the lung of *gabra4* CKO mice.

### GABRA4 is essential for activation of autophagy and phagosomal maturation of Mtb in macrophages

**Figure 3.**
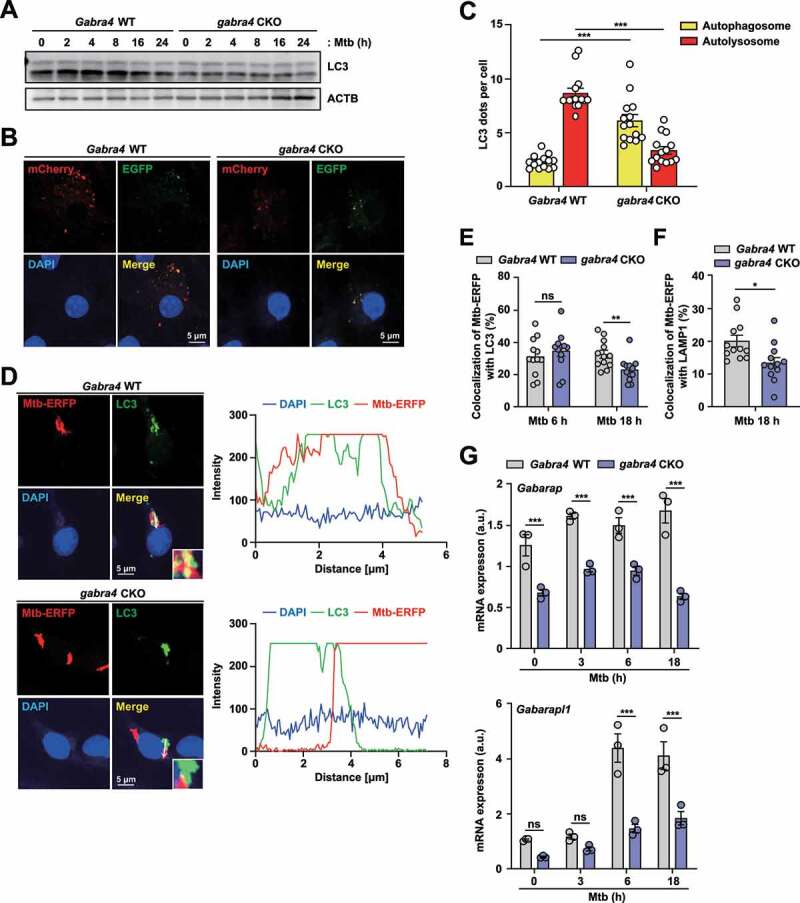
Regulation of autophagy and phagosomal maturation of Mtb by GABRA4 in macrophages. (**A**) Western blot of LC3 in Mtb-infected BMDMs from *Gabra4* WT and *gabra4* CKO mice. (**B, C**) *Gabra4* WT and *gabra4* CKO BMDMs transduced with retroviral pBABE-puro mCherry-EGFP-LC3B were infected with Mtb (MOI 5) and analyzed for LC3 dots by confocal microscopy. Shown are the representative images (B) and numbers of yellow (autophagosomes) and red (autolysosomes) LC3 dots per cell (C). Scale bar, 5 μm. (**D**) *Gabra4* WT and *gabra4* CKO BMDMs infected with Mtb-ERFP (MOI 5) for LC3 and DAPI (green and blue, respectively) staining. Representative confocal micrographs of Mtb-ERFP colocalization with LC3. Scale bar, 5 μm (left). Colocalization of Mtb-ERFP and LC3 are quantified and shown in graphs (right). (**E**) Quantitation of colocalization of Mtb-ERFP with LC3. (**F**) *Gabra4* WT and *gabra4* CKO BMDMs infected for 18 h with Mtb-ERFP (MOI 5) were stained with LAMP1. Quantitation of colocalization of Mtb-ERFP with LAMP1. (**G**) *Gabra4* WT and *gabra4* CKO PMs were infected with Mtb (MOI 1) for the indicated times and the expression levels of *Gabarap* and *Gabarapl1* were analyzed by qRT-PCR. Results shown are means ± SD (C, E–G). Two-tailed Student’s *t*-test (C, E, F) or two-way ANOVA (G) was used to assess significance. Data are representative of three independent experiments. *p < 0.05, **p < 0.01, ***p < 0.001; ns, not significant.

Although exogenous treatment of macrophages with GABA activates autophagy [3], it is unclear whether GABRA4 is involved in autophagy activation in response to mycobacterial infection. We thus infected *Gabra4* WT and *gabra4* CKO BMDMs with Mtb and examined the activation of autophagy. Mtb infection resulted in an increase in the LC3-II to LC3-I ratio, which represent increased processing of LC3A/B, in *Gabra4* WT BMDMs than in *gabra4* CKO BMDMs in a time-dependent manner (Fig. 3A). To further assess whether GABRA4 is involved in activating autophagic flux in macrophages, *Gabra4* WT and *gabra4* CKO BMDMs were transduced with a mCherry-enhanced green fluorescent protein (EGFP)-LC3B-expressing retrovirus before Mtb infection. The number of autolysosomes (acidic pH quenches GFP fluorescence), as marked by level of punctate structures in red (mCherry; acid-stable), was markedly suppressed in *gabra4* CKO BMDMs after Mtb infection than in *Gabra4* WT BMDMs (Fig. 3B and 3C). Notably, Mtb infection significantly increased the phagosomes and autophagosomes colocalization in *Gabra4* WT BMDMs after 6 and 18 h of infection, and this was significantly decreased in *gabra4* CKO BMDMs at 18 h after infection (Fig. 3D and 3E). Similarly, Mtb phagosomal colocalization with lysosomes was suppressed considerably in *gabra4* CKO BMDMs at 18 h after infection compared to *Gabra4* WT BMDMs (Fig. 3F). Together, these data strongly indicate that GABRA4 is required to activate autophagic flux and phagosomal maturation in macrophages during Mtb infection.

To investigate how GABRA4 is involved in autophagy during infection and inflammation, we performed qRT-PCR amplification of *Gabarap* and *Gabarapl1*, which are regulated by GABA-induced host defense in macrophages [3]. The expression of *Gabarap* and *Gabarapl1* was decreased significantly in *gabra4* CKO PMs after Mtb (Fig. 3G) or Mabc (Fig. S3A) infection compared to *Gabra4* WT PMs. Because the transcription factor FOXO3 mediates autophagy induction by transcriptional activation of several autophagy genes [33,34], we analyzed the mRNA levels of several ATGs. The *Foxo3a* and *Map1lc3a* mRNA levels were decreased significantly in *gabra4* CKO PMs compared to *Gabra4* WT PMs after infection with Mtb (Fig. S3B) or Mabc (Fig. S3C). Together, these data implicate GABRA4 in the autophagy gene induction, autophagy activation and phagosomal maturation of Mtb in macrophages during infection.

### GABRA4 is essential for the activation of autophagy via Ca^2+^-AMPK signaling in macrophages

**Figure 4.**
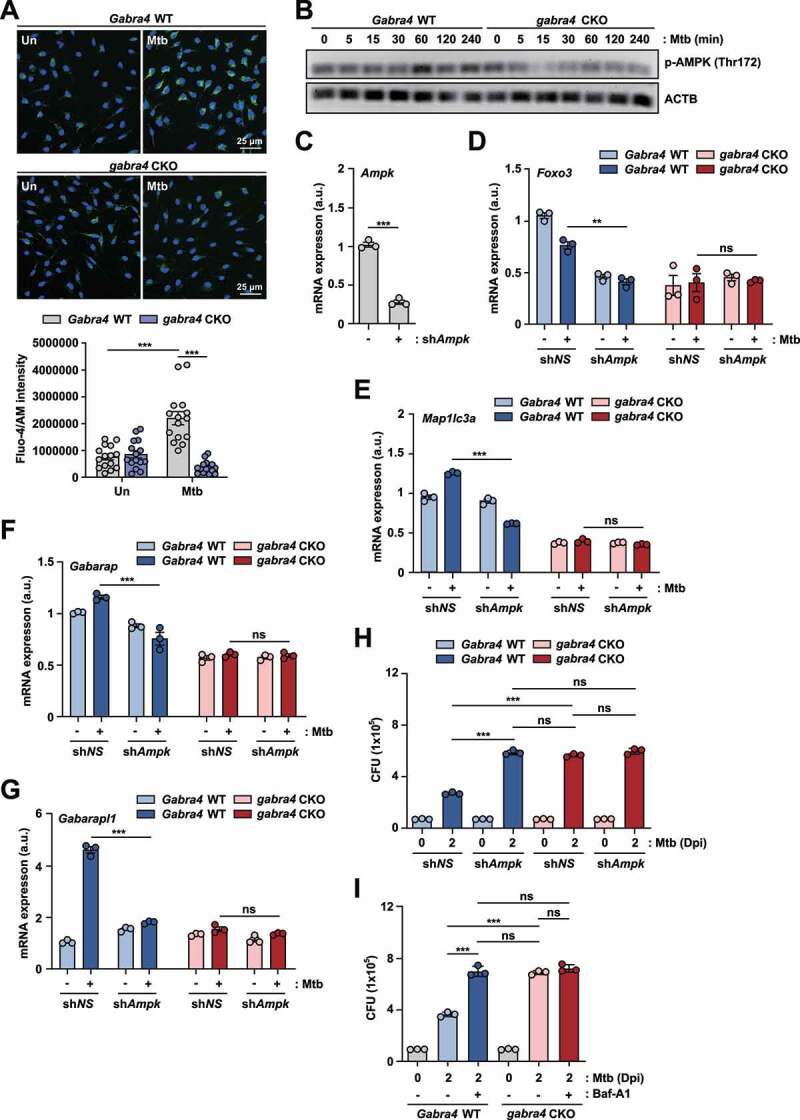
GABRA4 regulates autophagy via Ca^2+^-mediated AMPK signaling, which is necessary for antimicrobial responses, in macrophages. (**A**) BMDMs from *Gabra4* WT and *gabra4* CKO mice were incubated with Fluo-4/AM before infection with Mtb (MOI 5). Representative images. Scale bar, 25 μm (top). Quantitative data of Fluo-4/AM intensity (bottom). (**B**) *Gabra4* WT and *gabra4* CKO BMDMs infected with Mtb (MOI 3) for the indicated times were used to detect phospho-AMPK and ACTB levels by western blotting. (**C**) Confirmation of shRNA-mediated knockdown of *Ampk* in WT PMs transduced with sh*NS* or sh*Ampk*. (**D-G**) *Gabra4* WT and *gabra4* CKO PMs were transduced with sh*NS* or sh*Ampk*, as confirmed in (C), and infected for 18 h with Mtb (MOI 1) to measure the mRNA levels of *Foxo3* (D), *Map1lc3a* (E), *Gabarap* (F), and *Gabarapl1* (G). (**H, I**) Intracellular survival after Mtb infection (MOI 1) in *Gabra4* WT and *gabra4* CKO BMDMs transduced with sh*NS* or sh*Ampk* (H) and treated without or with bafilomycin A1 (Baf-A1, 200 nM) (I). Data are presented as means ± SD (A bottom, C-I). Two-tailed Student’s *t*-test (A bottom), unpaired *t*-test (C), or one-way ANOVA (D–I) was used to assess significance. Representatives of three independent experiments are shown. *p < 0.05, **p < 0.01, ***p < 0.001. Un, uninfected; Dpi, days post-infection; ns, not significant.

Given that signaling pathways involving intracellular Ca^2+^ flux and AMPK activation are critical for GABA-induced autophagy [3], we compared the intracellular Ca^2+^ flux between Mtb-infected *Gabra4* WT and *gabra4* CKO BMDMs. Mtb-induced intracellular Ca^2+^ release was significantly reduced in *gabra4* CKO BMDMs compared to *Gabra4* WT BMDMs (Fig. 4A). In addition, AMPK phosphorylation was significantly reduced in *gabra4* CKO BMDMs after Mtb infection compared to *Gabra4* WT BMDMs in a time-dependent manner (Fig. 4B). Given the role of GABRA4 in induction of autophagy genes, such as *Map1lc3a* and *Foxo3*, we investigated whether GABRA4-AMPK signaling is required for the expression of *Map1lc3a* and *Foxo3* in macrophages during Mtb infection. We used shRNA specific for AMPK (sh*Ampk*) to knockdown AMPK in macrophages and measured the mRNA levels of *Map1lc3a* and *Foxo3* in *Gabra4* WT and *gabra4* CKO PMs. Silencing of *Ampk* markedly suppressed the *Map1lc3a* and *Foxo3* mRNA levels in *Gabra4* WT PMs, but not in *gabra4* CKO PMs (Fig. 4C-E). Additionally, the *Gabarap* and *Gabarapl1* mRNA levels were significantly decreased in *Gabra4* WT PMs, but not in *gabra4* CKO PMs, by knockdown of *Ampk* (Fig. 4F and 4G). Together, these results suggest that AMPK activation is required for GABRA4-mediated expression of *Foxo3* and autophagy genes in macrophages during Mtb infection.

To further investigate the mechanistic link between GABRA4-mediated autophagy and antimicrobial responses, we examined if blockade of autophagy by silencing of *Ampk* or using a pharmacological inhibitor of autophagy affected GABRA4-induced antimicrobial responses against Mtb infection. As shown in Fig. 4H, *Ampk* knockdown significantly increased intracellular Mtb growth in *Gabra4* WT BMDMs, whereas sh*Ampk* did not affect bacterial growth in *gabra4* CKO BMDMs. In addition, bafilomycin A1, an inhibitor of lysosomal acidification, significantly increased intracellular Mtb survival in *Gabra4* WT BMDMs, but not in *gabra4* CKO BMDMs (Fig. 4I). These findings implicate AMPK in the link between GABRA4-induced autophagy and antimicrobial responses during Mtb infection. Because autophagy is a quality-control system that regulates inflammatory responses to pathogens or danger signals [19], we assessed whether GABRA4-mediated AMPK activation controls inflammatory cytokine responses during infection. Silencing of *Ampk* significantly increased Mabc-induced *Il1b* and *Il6* expression in *Gabra4* WT PMs but not in *gabra4* CKO PMs (Fig. S4A and S4B). Collectively, these data suggest involvement of the GABRA4-AMPK axis in the orchestration of autophagy, antimicrobial responses, and inflammatory responses in macrophages during infection.

### Myeloid GABRA4 is required for the maintenance of mitochondrial respiration and generation of the mitochondrial ROS that activate autophagy and host defense

**Figure 5.**
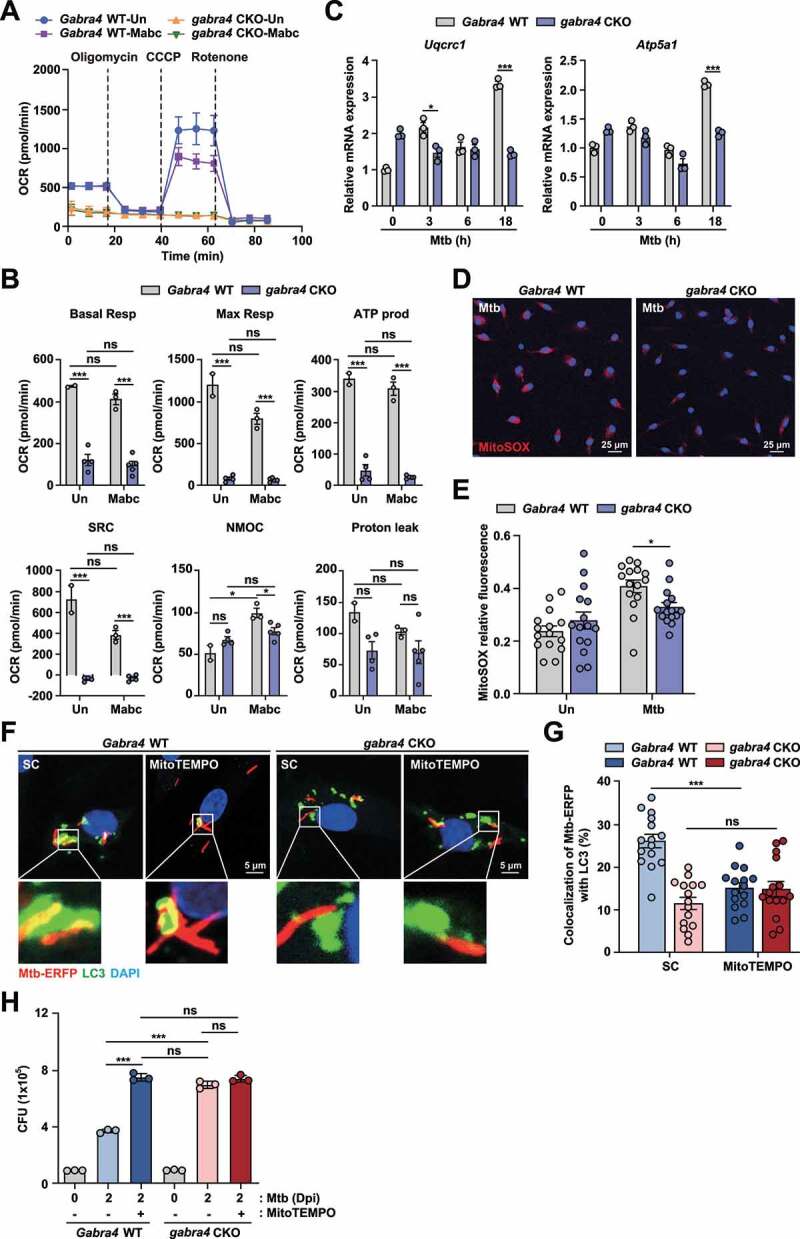
GABRA4 is required for mitochondrial respiration and mtROS generation, activating autophagy and antimicrobial responses during mycobacterial infection. (**A, B**) OCR profile (A) and parameters (B) in Mabc-infected PMs from *Gabra4* WT and *gabra4* CKO mice. (**C**) PMs from *Gabra4* WT and *gabra4* CKO mice were infected with Mtb (MOI 1), and the mRNA levels of *Uqcrc1* and *Atp5a1* were measured by qRT-PCR. (**D, E**) *Gabra4* WT and *gabra4* CKO BMDMs were infected with Mtb (MOI 5) for 4 h, before MitoSOX (red) and DAPI (for nuclei; blue) staining. (**D**) Representative images of MitoSOX staining (scale bar 25 μm) are shown. (**E**) Relative fluorescence of MitoSOX. (**F, G**) *Gabra4* WT and *gabra4* CKO BMDMs were pretreated with MitoTEMPO (50 μM) for 24 h, and cells were infected with Mtb-ERFP (MOI 5) for 18 h. (**F**) Representative images are shown. Scale bar, 5 μm. (**G**) Quantification of colocalization of Mtb-ERFP with LC3. (**H**) Intracellular survival after Mtb infection (MOI 1) in *Gabra4* WT and *gabra4* CKO BMDMs treated without or with MitoTEMPO (100 μM). Means ± SEM (B, C, E, and G) or ± SD (H). The two-tailed Student’s *t*-test (B, C, E, and G) or one-way ANOVA (H) was used to evaluate significance. Representative of three independent experiments. *p < 0.05 and ***p < 0.001. Un, uninfected; Dpi, days post-infection; CCCP, carbonyl cyanide 3-chlorophenylhydrazone; SC, solvent control. Basal Resp, basal respiration; Max Resp, maximal respiration; ATP prod, ATP production; SRC, spare respiratory capacity; NMOC, non-mitochondrial oxidative capacity; Proton leak, protoen leakage; ns, not significant.

Because autophagy is required for mitochondrial quality control and homeostasis [19], we next investigated whether GABRA4 is involved in regulating mitochondrial respiratory function and producing mtROS. To examine this, we performed a Seahorse assay to analyze bioenergetic signatures by measuring the oxygen consumption rate (OCR) which indicates oxidative phosphorylation, in *Gabra4* WT and *gabra4* CKO PMs after infection. The OCR profile was significantly reduced in *gabra4* CKO PMs than *Gabra4* WT PMs in response to Mabc infection (Fig. 5A), suggesting that *Gabra4* deficiency downregulates mitochondrial respiration. Notably, the levels of OCR parameters such as basal and maximal respiration, ATP production, spare respiratory capacity, and non-mitochondrial oxygen consumption were markedly downregulated in *gabra4* CKO PMs compared with *Gabra4* WT PMs after Mabc infection (Fig. 5B). Also, the expression of mitochondrial respiratory chain complex III (*Uqcrc1*) and complex V (*Atp5a1*) genes were significantly decreased in *gabra4* CKO PMs compared to *Gabra4* WT PMs after Mtb infection (Fig. 5C). Given that AMPK is an essential regulator of mitochondrial metabolism and mtOXPHOS [35], we assessed the effect of *Ampk* knockdown on *Uqcrc1* and *Atp5a1* expression. Mabc-mediated *Uqcrc1* and *Atp5a1* expression levels were significantly downregulated by *Ampk* knockdown in *Gabra4* WT BMDMs but were not modulated by sh*Ampk* in *gabra4* CKO BMDMs (Fig. S5A and S5B).

Because complex III of the mitochondrial respiratory chain is essential for the production of mtROS [36,37], we examined whether the level of mtROS varies between *Gabra4* WT and *gabra4* CKO BMDMs during infection. As shown, mtROS level was significantly lower in *gabra4* CKO BMDMs after 4 h of Mtb infection than in *Gabra4* WT BMDMs (Fig. 5D and 5E). mtROS is essential for autophagy activation in response to diverse stimuli [38], so we evaluated whether GABRA4-mediated mtROS generation is necessary for autophagy activation and antimicrobial responses to Mtb infection. In the presence of the mtROS scavenger MitoTEMPO, colocalization of Mtb phagosomes with LC3 autophagosomes and antimicrobial responses were significantly downregulated in *Gabra4* WT BMDMs (Fig. 5F-H). However, MitoTEMPO did not attenuate Mtb-contained autophagosome formation and antimicrobial host defense in *gabra4* CKO BMDMs (Fig. 5F-H). Taken together, the above findings suggest that GABRA4-AMPK signaling is essential for the maintenance of mtOXPHOS and mtROS generation, which is necessary for autophagy activation and antimicrobial host defense in macrophages during infection.

### Activation of GABA_A_R signaling leads to antimicrobial responses in macrophages via autophagy activation

**Figure 6.**
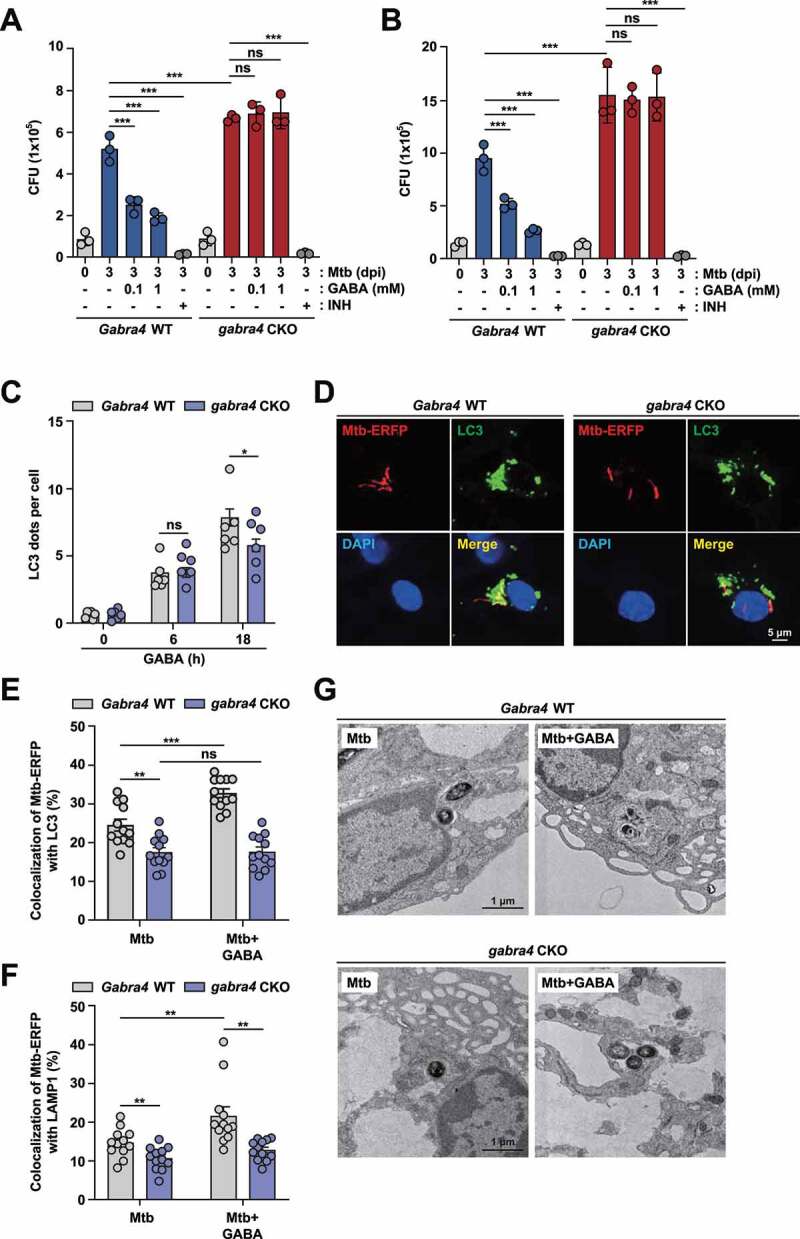
GABRA4 signaling is essential for GABA-mediated antibacterial autophagy in macrophages. (**A, B**) Intracellular survival of Mtb (MOI 1) after 3 days in BMDMs (A) and PMs (B) from *Gabra4* WT and *gabra4* CKO mice in the presence of GABA or isoniazid (INH, 0.5 μg/mL). (**C**) *Gabra4* WT and *gabra4* CKO BMDMs treated with GABA (100 μM) were stained for LC3. The number of LC3 dots per cell is shown. (**D-F**) BMDMs from *Gabra4* WT and *gabra4* CKO mice were infected with Mtb-ERFP (MOI 5) in the presence of GABA (100 μM) for 18 h, and stained with LC3 (D, E) or LAMP1 (F). (**D**) Confocal micrographs. Scale bar, 5 μm. (**E**) Quantification of colocalization of Mtb-ERFP with LC3. (**F**) Quantification of colocalization of Mtb-ERFP with LAMP1. (**G**) *Gabra4* WT and *gabra4* CKO BMDMs after infection with Mtb (MOI 5) and incubation with GABA (100 μM) for 18 h were subjected to transmission electron microscopy (TEMs). Representative transmission electron micrographs are shown. Scale bar, 1 μm. Means ± SEM (A-C, E, and F). One-way ANOVA (A-C) and two-tailed Student’s *t-*test (E, F) were used to evaluate significance. Representative of three independent experiments are shown. *p < 0.05, **p < 0.01, ***p < 0.001. ns, not significant; dpi, days post-infection; GABA, γ-aminobutyric acid; INH, isoniazid.

Because GABA inhibits intracellular mycobacterial growth in macrophages [3], we next assessed whether GABA-mediated antimicrobial responses are mediated by GABRA4 in macrophages. To examine this, BMDMs or PMs were infected with Mtb and treated with increasing concentrations of GABA for 3 days, and a CFU assay was performed. As shown in Fig. 6A and 6B, GABA dose-dependently suppressed the intracellular bacterial growth, and this was significantly abrogated in *gabra4* CKO BMDMs (Fig. 6A) or PMs (Fig. 6B), suggesting that GABA-mediated antimicrobial host defense is dependent on the expression of GABRA4 in macrophages. Isoniazid (INH), an antibiotic for human tuberculosis, was used as a positive control. We assessed whether another GABA_A_R agonist activates antimicrobial responses. As shown in Fig. S6A, THIP (4,5,6,7-tetrahydroisoxazolo[5,4-c]pyridin-3-ol, also known as gaboxadol), a δ subunit-preferring GABA_A_R agonist [39], significantly attenuated intracellular growth of Mtb and BCG in *Gabra4* WT PMs (Mtb at 10 and 100 μM; BCG at 100 μM). These results suggest that agonists of GABA_A_R promote antimicrobial responses in macrophages.

We further examined whether GABA-induced autophagy activation depends on GABRA4 expression. For this, *Gabra4* WT and *gabra4* CKO BMDMs were incubated with GABA, and activation of autophagy was determined by measuring the formation of LC3-II-positive structures, an indicator of the absolute number of autophagosomes. As reported previously [3], GABA significantly activated the formation of LC3A/B puncta in *Gabra4* WT BMDMs; however, GABA-induced autophagosome formation was significantly decreased in *gabra4* CKO BMDMs (Fig. 6C). In addition, GABA treatment of *Gabra4* WT BMDMs increased colocalization of Mtb with LC3 autophagosomes and lysosomes, which was markedly abrogated in *gabra4* CKO BMDMs (Fig. 6D and 6E for autophagosomes and Fig. 6F for lysosomes). Similar findings were observed in *Gabra4* WT and *gabra4* CKO PMs (Fig. S6B and S6C, for autophagosomes and lysosomes, respectively). Moreover, ultrastructural analysis revealed that GABA treatment of *Gabra4* WT BMDMs significantly increased the number of autophagosomal and autolysosomal structures, compared with *gabra4* CKO BMDMs (Fig. 6G). Therefore, GABA-induced antibacterial autophagy is dependent on GABRA4 expression in macrophages.

### GABRA4 is associated with the maintenance of aerobic glycolysis and is required to maintain the concentrations of glycolysis-related metabolites

**Figure 7.**
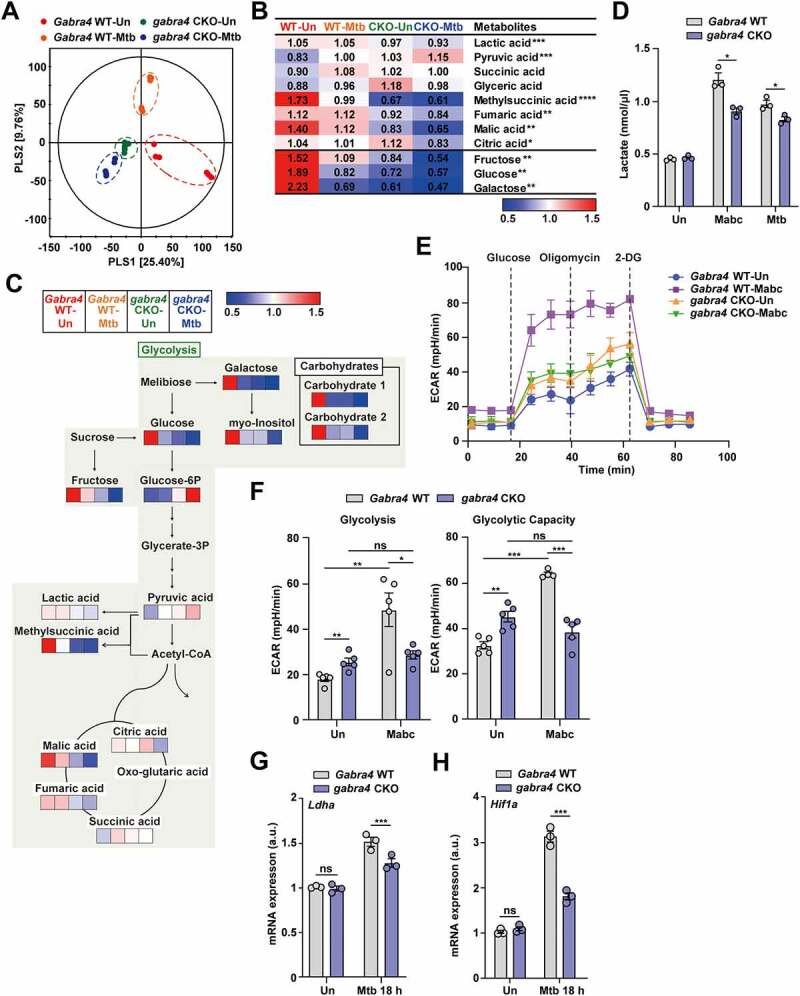
GABRA4 is required for glucose metabolism and optimal aerobic glycolysis in macrophages. (**A**) PLS-DA score plots acquired from non-targeted metabolite profiling of macrophages from *Gabra4* WT and *gabra4* CKO mice according to Mtb infection analyzed by GC-TOF/MS (VIP > 1.0, *p* < 0.05). (**B**) Heatmap analysis of the concentrations of glycolysis-related metabolites and amino acids. Numbers show the fold changes by an average peak area of each metabolite. Blue and red, metabolites significantly decreased and increased, respectively. Asterisks indicate significantly different metabolites. (**C**) Metabolic pathways were analyzed based on differentially expressed metabolites. Colored squares (blue-to-red) depict fold changes normalized to the experimental group. (**D**) Extracellular lactate levels in *Gabra4* WT and *gabra4* CKO PMs infected for 18 h with Mabc (MOI 3) or Mtb (MOI 3). (**E, F**) Extracellular acidification profile (ECAR; E) and various parameters (F) in Mabc-infected (18 h) *Gabra4* WT and *gabra4* CKO PMs. (**G, H**) *Ldha* (G) and *Hif1a* (H) mRNA levels in Mtb (MOI 1)-infected *Gabra4* WT and *gabra4* CKO PMs. Means ± SEM are shown (B, D, F-H). One-way ANOVA (B), two-tailed Student’s *t*-test (D), or two-way ANOVA (F-H) was used to evaluate significance. Representative of three independent experiments are shown. *p < 0.05, **p < 0.01, ***p < 0.001, and ****p < 0.0001. ns, not significant; Un, uninfected; 2-DG, 2-deoxy-glucose.

Immune-modulating metabolites regulate functional responses of immune cells, such as dendritic cells and macrophages [40,41]. To investigate immune-related metabolite changes in macrophages following *Gabra4* deficiency and Mtb infection, using GC-TOF/MS, we conducted a metabolomic analysis of Mtb-infected *Gabra4* WT and *gabra4* CKO macrophages. A partial least squares-discriminant analysis (PLS-DA) model revealed significant segregation of each sample by *Gabra4* deficiency and Mtb infection (Fig. 7A). In particular, *Gabra4* deficiency had a more significant effect on the metabolite profiles of macrophages than Mtb infection. To identify the differentially expressed metabolites by *Gabra4* genotype and Mtb infection, we analyzed the variable importance in projection (VIP) scores (> 1.0) by PLS-DA. Between the experimental groups, there were significant differences in a total of 18 metabolites—including organic acids, sugars, sugar alcohols, and others (Fig. S7A). Additionally, other organic acids related to aerobic glycolysis (succinic acid, glyceric acid, methyl succinic acid, and citric acid) were analyzed by targeted metabolomics (VIP scores < 1.0). Heatmap and pathway analysis showed that the aerobic glycolysis-related metabolites were significantly depleted by *Gabra4* deficiency irrespective of Mtb infection (Fig. 7B and 7C). Further, the relative concentrations of metabolites were compared using one-way ANOVA and Tukey’s multiple comparison test (Fig. S7B). Also, the extracellular lactate level was significantly lower in Mabc- or Mtb-infected *gabra4* CKO PMs than *Gabra4* WT PMs (Fig. 7D). The results showed that *Gabra4* deficiency alters the concentrations of glycolysis-associated metabolites.

To validate the involvement of GABRA4 in the regulation of aerobic glycolysis during infection, a glycolysis stress test was performed to monitor the extracellular acidification rate (ECAR). The glycolysis parameter following the addition of glucose, glycolytic capacity after adding oligomycin (Fig. 7E and 7F), and non-glycolytic acidification (Fig. S7C) were increased by Mabc-infected *Gabra4* WT PMs but were significantly lower in *gabra4* CKO PMs. Metabolomics analysis showed that the glucose level was significantly lower in Mtb-infected *gabra4* CKO macrophages than in *Gabra4* WT macrophages. Therefore, we measured the expression level of glucose transporter 1 (*Glut1*) in Mtb-infected *Gabra4* WT and *gabra4* CKO PMs. The mRNA level of *Glut1* was significantly decreased in Mtb-infected *gabra4* CKO PMs compared to *Gabra4* WT PMs (Fig. S7D). Additionally, we evaluated if hypoxia-inducible factor (HIF)-1α, an essential metabolic target during the glycolytic switch, and its downstream target lactate dehydrogenase isoform A (LDHA), are affected by *Gabra4* deficiency. The *Ldha* and *Hif1a* mRNA levels were significantly decreased in Mtb-infected *gabra4* CKO macrophages compared to *Gabra4* WT macrophages (Fig. 7G and 7H). These findings suggest that GABRA4 is required for functional aerobic glycolysis and for maintaining the concentrations of glycolysis-related metabolites in macrophages.

### Myeloid GABRA4 is essential for controlling proinflammatory responses and NLRP3 inflammasome activation *in vivo* and *in vitro*

**Figure 8.**
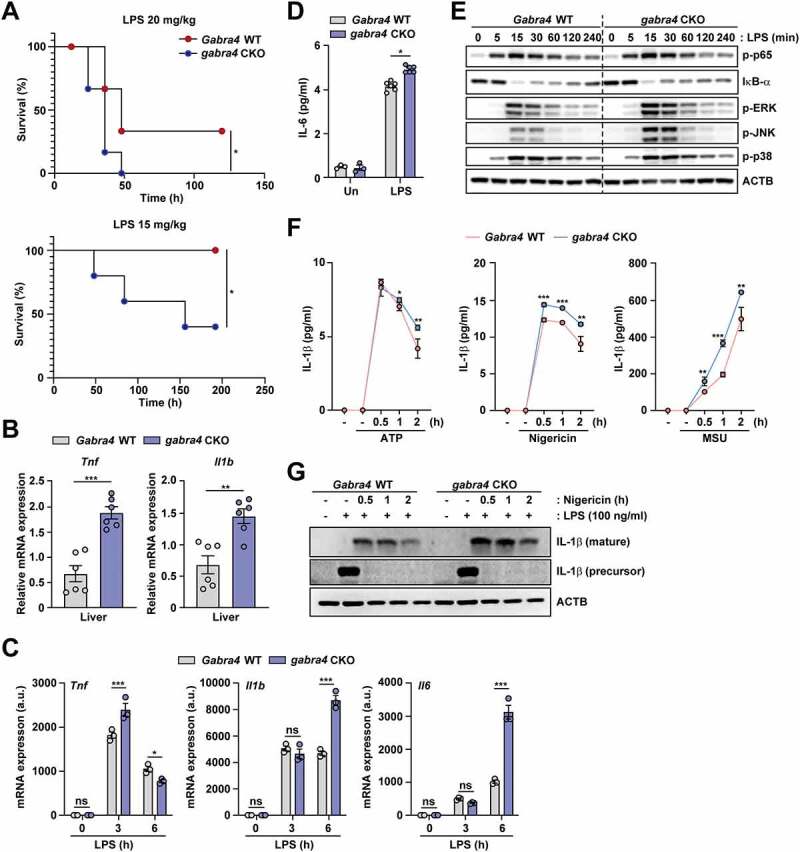
Myeloid GABRA4 is essential for controlling excessive proinflammatory responses *in vivo* and *in vitro*. (**A**) Survival of *Gabra4* WT and *gabra4* CKO mice injected with LPS (15 or 20 mg/kg, i.p.). (**B**) *Tnf* and *Il1b* mRNA levels in the livers of *Gabra4* WT and *gabra4* CKO mice injected with LPS (14 mg/kg, i.p.) for 6 h. (**C**) qRT-PCR of *Tnf, Il1b*, and *Il6* mRNA in *Gabra4* WT and *gabra4* CKO BMDMs stimulated with LPS (100 ng/mL). (**D**) ELISA of IL-6 in supernatant after 18 h of LPS (100 ng/mL) stimulation in *Gabra4* WT and *gabra4* CKO BMDMs. (**E**) Western blot of phosphorylation of NF-κB and MAPK (ERK, p38, and JNK) in LPS (100 ng/mL) stimulated *Gabra4* WT and *gabra4* CKO BMDMs. (**F**) Levels of IL-1β in supernatants from *Gabra4* WT and *gabra4* CKO PMs sensitized with LPS (100 ng/mL) and stimulated with ATP (5 mM), nigericin (10 μM), and MSU (200 μg/mL). (**G**) The level of mature IL-1β determined by western blotting of supernatant from LPS (100 ng/mL)-sensitized and nigericin (10 μM)-stimulated *Gabra4* WT and *gabra4* CKO PMs. IL-1β (precursor) and ACTB were detected in the cell lysates. Means ± SEM are shown (B-D, and F). The log-rank (Mantel–Cox) test (A), two-tailed Student’s *t*-test (B, D, F), or two-way ANOVA (C) was used to evaluate significance. Data are representative of three independent experiments. *p < 0.05, **p < 0.01, ***p < 0.001. ns, not significant; Un, untreated; MSU, monosodium urate.

In order to obtain insight into the role of GABRA4 in innate immunity, we used a murine model of LPS-induced septic shock to examine the survival of *Gabra4* WT and *gabra4* CKO mice. During the several days of observation after intraperitoneal (i.p.) injection of LPS, *gabra4* CKO mice had increased lethality compared to *Gabra4* WT mice (Fig. 8A). In addition, the liver and spleen tissues from *gabra4* CKO mice showed enhanced expression of proinflammatory cytokines compared to *Gabra4* WT mice (Fig. 8B and Fig. S8A). We next examined LPS-induced inflammatory cytokine expression in macrophages from *Gabra4* WT and *gabra4* CKO mice. The mRNA expressions and protein levels of inflammatory cytokines were significantly increased in *gabra4* CKO BMDMs and PMs, compared to those from *Gabra4* WT mice, at 3 and 6 h (mRNA; Fig. 8C in BMDMs and Fig. S8B in PMs) and 18 h (ELISA; Fig. 8D) after LPS stimulation. In addition, LPS-induced phosphorylation of MAPKs (p-ERK1/2 and p-JNK), but not nuclear factor (NF)-κB, was substantially higher in *gabra4* CKO BMDMs than in *Gabra4* WT BMDMs (Fig. 8E).

The NOD-, LRR- and pyrin domain-containing protein (NLRP) 3 inflammasome consists of NLRP3, the adaptor apoptosis-associated speck-like protein containing a CARD (ASC), and pro-caspase-1, and is necessary for maturation of pro-IL-1β into IL-1β, playing a role in the pathogenesis of several inflammatory diseases [42-44]. To ascertain if GABRA4 is involved in NLRP3 inflammasome activation, *Gabra4* WT and *gabra4* CKO PMs were sensitized with LPS (100 ng/mL) for 4 h before stimulating with several inflammasome stimuli, namely ATP (5 mM), nigericin (10 μM), or monosodium urate (MSU) (200 μg/mL). The level of IL-1β in cell culture supernatant was measured by ELISA, and that of the mature form of IL-1β in supernatant by western blotting. LPS/ATP, LPS/nigericin, and LPS/MSU induced more secretion of IL-1β in *gabra4* CKO PMs than *Gabra4* WT PMs in a time-dependent manner (Fig. 8F). In parallel, the level of mature IL-1β was higher in supernatant from LPS/nigericin-induced *gabra4* CKO macrophages than from *Gabra4* WT macrophages (Fig. 8G). These data indicate that myeloid GABRA4 expression is required for controlling exaggerated inflammatory cytokine signaling and NLRP3 inflammasome pathway activation.

### GABRA4 mediates activation of autophagy in macrophages in response to inflammatory stimuli via Ca^2+^-mediated AMPK signaling

**Figure 9.**
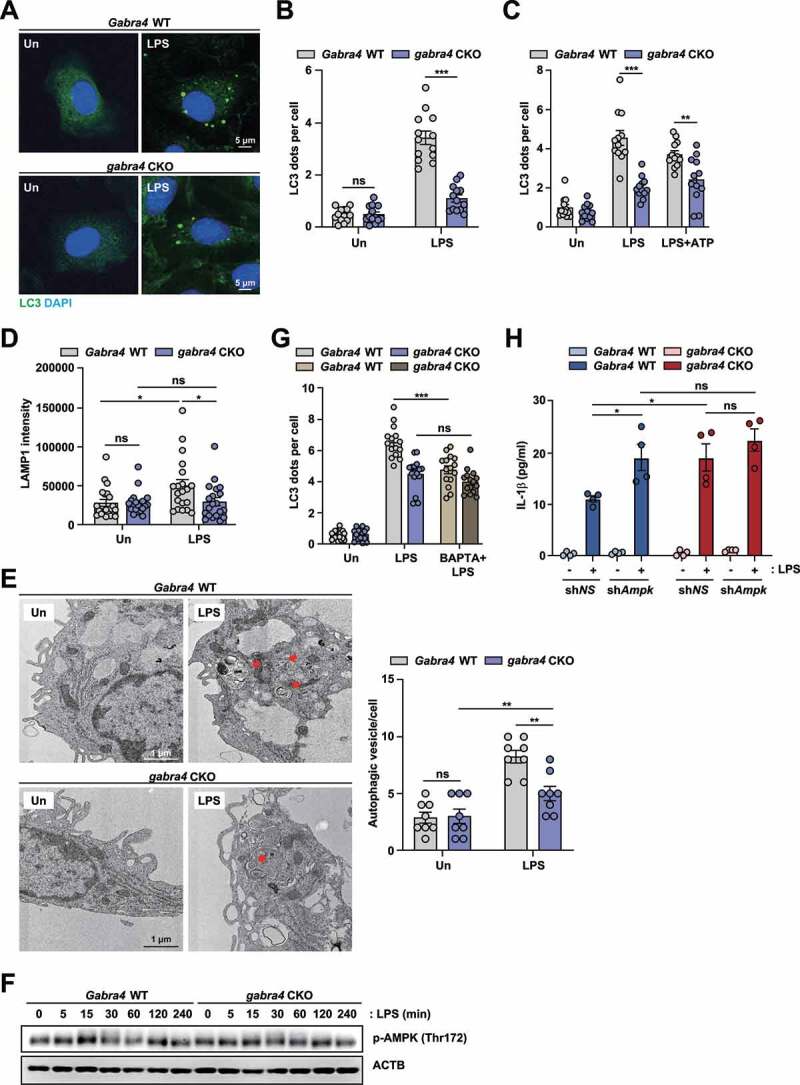
GABRA4 signaling contributes to LPS-induced autophagy activation via the Ca^2+^-AMPK pathway in macrophages. (**A, B**) *Gabra4* WT and *gabra4* CKO BMDMs were stimulated with LPS (100 ng/mL) for 6 h and stained with LC3 (green) and DAPI (for nuclei; blue). (**A**) Representative microscopic images are shown. Scale bar, 5 μm. (**B**) Quantitative results of LC3 dots per cell. (**C**) BMDMs from *Gabra4* WT and *gabra4* CKO mice were incubated with LPS (100 ng/mL) for 4 h and stimulated without or with ATP (5 mM) for 30 min before staining with LC3. Quantitative data of LC3 dots per cell. (**D**) BMDMs from *Gabra4* WT and *gabra4* CKO mice were stimulated for 6 h with LPS (100 ng/mL), and cells were stained with LAMP1. Quantitative data of LAMP1 intensity. (**E**) *Gabra4* WT and *gabra4* CKO PMs were treated with LPS (100 ng/mL) for 6 h. Representative TEMs of PMs. Scale bar, 1 μm (left). Quantitative data of autophagic vesicle numbers (right). (**F**) Western blot analysis of phospho-AMPK and ACTB in *Gabra4* WT and *gabra4* CKO PMs stimulated with LPS (100 ng/mL). (**G**) LC3 (green) staining of *Gabra4* WT and *gabra4* CKO BMDMs treated with BAPTA-AM (BAPTA, 25 μM) and stimulated with LPS (100 ng/mL); DAPI (blue) shows nuclei. Quantitative data of LC3 dots per cell. (**H**) sh*NS* or sh*Ampk* transduced *Gabra4* WT or *gabra4* CKO BMDMs were stimulated for 18 h with LPS (100 ng/mL) and the level of IL-1β was determined. Means ± SEM (B–E right, G, and H). The two-tailed Student’s *t*-test (B-E right, G, and H) was used to evaluate significance. Representatives of three independent experiments are shown. *p < 0.05, **p < 0.01, ***p < 0.001. Un, uninfected; ns, not significant.

Given the essential role of GABRA4 in autophagy activation during Mtb infection, we assessed the function of GABRA4 in the activation of LPS-induced autophagy. As shown, formation of LC3A/B puncta was markedly decreased in *gabra4* CKO BMDMs in response to LPS or NLRP3 inflammasome stimuli (LPS plus ATP), as compared to *Gabra4* WT BMDMs (Fig. 9A-C) and PMs (Fig. S9A and S9B). In addition, LPS-induced LAMP1 intensity was significantly suppressed in *gabra4* CKO BMDMs compared to *Gabra4* WT BMDMs (Fig. 9D). Moreover, the quantity of autophagosomal and autolysosomal structures were significantly decreased in *gabra4* CKO PMs than in *Gabra4* WT PMs after LPS stimulation (Fig. 9E), as revealed by ultrastructural analysis. In addition, we tested whether LPS-induced autophagy depends on GABRA4-Ca^2+^-AMPK signaling in macrophages. Similar to the findings in Mtb infection, intracellular Ca^2+^ release (Fig. S9C) and AMPK phosphorylation (Fig. 9F) were reduced significantly in *gabra4* CKO macrophages in comparison to *Gabra4* WT macrophages in response to LPS stimulation.

To determine whether intracellular Ca^2+^ signaling contributes to the LPS-triggered autophagy in macrophages and whether it depends on GABRA4 expression, we pretreated *Gabra4* WT and *gabra4* CKO BMDMs with BAPTA-AM, an intracellular calcium chelator, and analyzed autophagy induction. In *Gabra4* WT BMDMs, BAPTA-AM significantly reduced the number of LPS-induced LC3 punctate formation; however, it had no effect on autophagosome formation in *gabra4* CKO BMDMs (Fig. 9G). Further, we examined whether GABRA4-mediated AMPK activation modulates inflammatory-cytokine gene expression in response to LPS. Silencing of *Ampk* significantly increased the LPS-induced IL-1β level in *Gabra4* WT, but not in *gabra4* CKO, BMDMs (Fig. 9H). Collectively, these findings imply that Ca^2+^-AMPK signaling is needed for GABRA4-mediated autophagy activation and regulation of inflammation.

## Discussion

**Figure 10.**
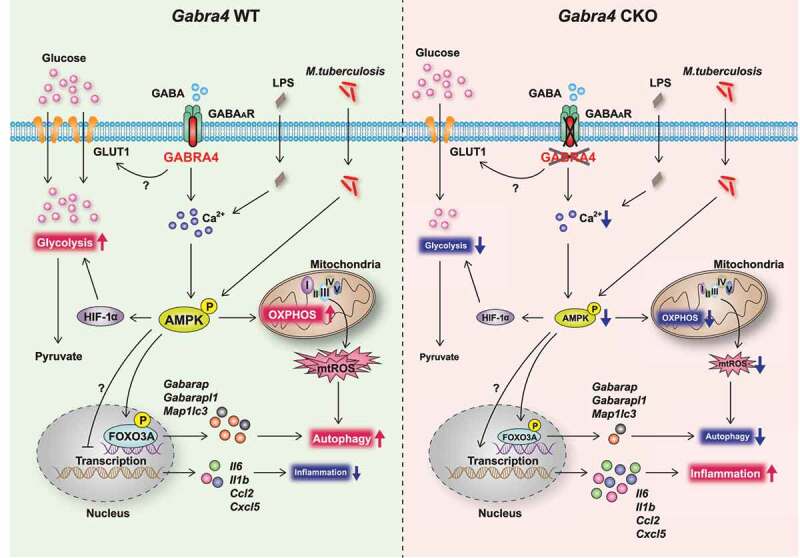
Schematic of the role of GABRA4 in modulating innate immune responses. GABRA4 activates autophagy from transcriptional level to autophagic flux via the Ca^2+^-AMPK-FOXO3A pathway, thereby inhibiting inflammation in response to pathogenic or dangerous stimuli. GABRA4-mediated AMPK activation is critical for the orchestration of autophagy, inflammatory responses, mtOXPHOS, and antimicrobial responses. In addition, GABRA4 is required for the maintenance of aerobic glycolysis via AMPK-dependent HIF-1α signaling. Therefore, GABRA4, via Ca^2+^-AMPK signaling, coordinates autophagy, inflammation, and innate host defenses.

We evaluated the significance of GABRA4 signaling in host defense in macrophages during infection and inflammation. The data show that myeloid GABRA4 signaling is crucial for the activation of autophagy, a cell-autonomous pathway for innate defense and controlling inflammation [19,23]. GABRA4-mediated autophagy signaling involves intracellular calcium release and the AMPK pathway, which trigger the expression of *Gabarap, Gabarapl1*, and *foxo3*—critical components of the autophagy network. Also, GABRA4 signaling is necessary to maintain mtOXPHOS and mtROS production, thereby activating autophagy and antimicrobial responses to mycobacterial infection. Furthermore, *Gabra4*-deficient macrophages had diminished glycolysis and lactate formation, as well as defective amino acid and sugar metabolism. Our data demonstrate that GABRA4 signaling, via AMPK pathway activation, orchestrates autophagy, antimicrobial host defense, inflammation, and immunometabolism in macrophages during infection and inflammation (Fig. 10).

GABA_A_Rs assembled of heteropentamers are highly structurally complex, and comprise at least 19 subunits differentially expressed depending on the cell or tissue [45]. Indeed, GABRA4 is expressed in macrophages, monocytes, and dendritic cells, as well as neuronal cells [3,46-48]. Although GABRA4 in neuronal cells inhibits neurotransmission at synapses and outside of the synapse [49,50], the extraneuronal function of GABRA4 is unclear. Upon infection with the intracellular protozoan *Toxoplasma gondii*, dendritic cells possessing functional GABA_A_R exert migratory and chemotactic responses, promoting parasite dissemination into the central nervous system [51]. In addition, natural killer cells function as GABAergic cells and express GABA_A_R subunits during infection with *T. gondii*. Exogenous GABA affects the cytotoxic function of natural killer cells, enhancing the migratory responses of dendritic cells in the context of *T. gondii* infection [52]. During infection with coccidian parasites including *T. gondii* and *Neospora caninum*, gene silencing of the α4 subunit or β3 and ρ1 subunits significantly reduces hypermotility of human and murine dendritic cells, respectively [46]. Several peripheral innate immune cells are GABAergic, although there is little information on the function of immune cell-expressed GABRA4 in regulation of innate immunity during infection and inflammation. We reported that *Gabra4* is the dominant subtype of GABA_A_R in BMDMs [3]. In this study, we found that GABRA4 is critical for antimicrobial responses, despite its low expression level, in PMs and AMs. Therefore, GABRA4 in the peripheral immune system is critical for antimicrobial host responses. Moreover, depletion of macrophages by clodronate-liposomes significantly abrogated the differences in antimicrobial responses *in vivo* between *Gabra4* WT and *gabra4* CKO mice, suggesting the importance of macrophages for GABRA4-mediated host defense during mycobacterial infection.

Intracellular Ca^2+^ release and AMPK signaling activation contributed to GABRA4-dependent autophagy activation in macrophages. These findings partially support our previous report that GABA-mediated autophagy activation is mediated by intracellular Ca^2+^ flux in macrophages [3]. Extracellular Ca^2+^ influx, voltage-dependent calcium channel (VDCC) Cav1.3, and Na-K-Cl cotransporters (NKCCs) are interlinked with GABA_A_R signaling in phagocytes, influencing the *T. gondii*- and *N. caninum*-mediated increased motility of dendritic cells [46]. However, how GABA_A_R signaling activates autophagy in myeloid cells is unclear. Our data show that the Ca^2+^-AMPK pathway is required for transcriptional activation of the essential autophagy genes *Gabarap, Gabarapl1, Map1lc3a*, and *Foxo3* in the context of myeloid-specific *Gabra4* signaling during infection. FOXO3 activation is critical for various cellular pathways, including autophagy, mitochondrial function, and detoxification of oxidative stresses in a context-dependent manner [53-55]. Given that FOXO3 directly regulates autophagy gene networks [56,57] and expression of *Gabarap* and *Gabarapl1* [58], and that the activation of AMPK leads to a FOXO3A-dependent increase in the ATG protein level in primary myotubules [34], it is proposed that GABRA4-mediated FOXO3 signaling is critical for macrophage autophagy activation to maintain intracellular homeostasis during infection and inflammation. Combined with the fact that the GABARAP protein family is essential for the intracellular transport of GABA_A_R [59], our data strongly suggest that cross-talk between GABRA4 subunits and LC3/GABARAP family members is implicated in intracellular trafficking of GABA_A_R and autophagy.

Our data indicate that GABRA4 signaling is required for controlling excessive inflammatory responses to intracellular pathogens and inflammatory stimuli. Dysregulated anti-inflammatory switch in myeloid *Gabra4* deficiency increased mortality after LPS challenge in *gabra4* CKO mice compared with *Gabra4* WT mice. Therefore, GABRA4-mediated signaling is required to resolve macrophage and systemic inflammatory responses, control inflammasome complex activation, and reduce mortality. *Gabra4* global KO mice show increased eosinophilic lung infiltration and airway inflammation [47]. In addition, GABRA4 is abundantly expressed in human bronchial 16HBE cells and human pulmonary epithelial cells and is required to ameliorate pulmonary fibrosis [60]. Combined with those previous results, these data indicate that myeloid GABRA4 is a critical regulator of proinflammatory cytokine and chemokine production in response to LPS or NLRP3 inflammasome stimuli. However, *gabra4* CKO AMs do not control inflammatory responses to mycobacteria. Importantly, pulmonary neutrophil infiltration was significantly increased in *gabra4* CKO lungs during infection, and depletion of neutrophils markedly reduced the *in vivo* bacterial loads in the lung of *gabra4* CKO mice. These data suggest that increased neutrophil infiltration contributes to pathological inflammatory responses in the lungs of *gabra4* CKO mice during infection. Several issues remain to be clarified, such as the signal that regulates GABRA4 expression in the context of host defense and how GABA_A_R subunit proteins assemble to cooperate with GABRA4. In neuroblastoma cells, Gabra4 expression is controlled by the transcription factor early growth response 1 (EGR-1) [61]. EGR3-mediated upregulation of *GABRA4* promoter activity and the GABRA4 level is associated with epileptogenesis [62]. Future studies are warranted to clarify how GABRA4 expression and assembly are modulated in macrophages in the context of infection and inflammation.

We also addressed the role of GABRA4 in the elevation of mitochondrial respiration and ATP synthesis in macrophages during infection and inflammation. We found that myeloid deficiency of *Gabra4* markedly suppressed mitochondrial respiration and decreased mtROS generation, which affected autophagy, during infection. Importantly, scavenging of mtROS suppressed the control of intracellular Mtb survival in WT BMDMs, but not in *gabra4* CKO BMDMs. Given that mtROS directly activates classical macrophages and antibacterial responses [63], our data suggest that GABRA4-mediated mtROS contributes to antibacterial host defense by activating autophagy and macrophages. Moreover, GABRA4-mediated AMPK signaling links autophagy, inflammation, and immunometabolism. Indeed, silencing of AMPK significantly suppressed autophagy genes, but increased inflammatory cytokine responses in WT BMDMs, whereas it did not affect autophagy or inflammatory gene expression in *gabra4* CKO BMDMs, during Mtb infection. In addition, Mabc-mediated *Uqcrc1* and *Atp5a1* expression was significantly downregulated by *Ampk* knockdown in WT macrophages, but not in *Gabra4*-deficient macrophages. Given that AMPK is a key mediator of energy metabolism and mitochondrial homeostasis [35], our data suggest that the GABRA4-AMPK axis orchestrates antimicrobial host defense by modulating autophagy, inflammation, and mitochondrial respiration during infection and inflammation.

The data also revealed that *Gabra4* deficiency leads to diminished glycolysis and lactate formation, as well as defective sugar metabolism. An immunometabolic shift to aerobic glycolysis contributes to bacterial clearance in macrophages [64,65]. Because exogenous lactate treatment of macrophages upregulates antimicrobial responses against Mtb infection [66], the diminished lactate production in *Gabra4*-deficient macrophages might be associated with decreased antimicrobial host defense. We reported that HIF1A, which is regulated by mtROS, is required to activate inflammatory responses and xenophagy during mycobacterial infection [67]. Therefore, the HIF1A-LDHA pathway, regulated by GABRA4 signaling, is involved in activating autophagy and antimicrobial host defense during infection. The reason why *Gabra4*-deficient macrophages are defective in glucose and amino acid metabolism is not clear. Because GABA_A_R is a ligand-gated chloride channel [11], it is possible that dysregulated ionic transport (e.g., Na^+^ and/or Ca^2+^) due to GABA_A_R functional abnormalities affects glucose and amino acid transport and metabolism. *Glut1* expression was downregulated in *Gabra4*-deficient compared to WT macrophages. Given that GABA upregulates *GLUT4* expression in the liver [68], functional GABA_A_R may contribute to GLUT1 expression/activity in macrophages. Further investigation is warranted to identify the molecular mechanisms by which GABA_A_R signaling modulates glucose transport and/or metabolism in macrophages during infection and inflammation.

Collectively, our data indicate that GABRA4-AMPK signaling orchestrates innate host defense by coordinating autophagy, inflammation, and mitochondrial homeostasis (Fig. 10). A balance between innate immunity, inflammatory, and metabolic responses is crucial for eliminating invading pathogens and preventing damage from pathologic inflammation during infection. In this context, identifying myeloid GABRA4 as a critical regulator of autophagy may contribute to the development of novel host-directed therapeutics against infectious and inflammatory diseases.

## Materials and Methods

### Mice

*Gabra4*^fl/fl^ mice and LysM-Cre mice were generously provided by Dr. Chanki Kim (Center for Cognition and Sociality, Institute for Basic Science) and Dr. Chul-Ho Lee (Korea Research Institute of Bioscience and Biotechnology), respectively. Mice were kept under specific pathogen-free conditions, with a 12 h light/dark cycle maintained throughout. Littermate animals from heterozygous breeding pairs were established, and sex- and age-matched (6–8 weeks) mice were used for each experiment. Genomic DNA from *Gabra4* WT and *gabra4* CKO mice was prepared and analyzed by semiquantitative PCR reaction using Prime Taq Premix (GeNet Bio, G-3000). The sequences of the genotyping primers were as follows: forward, 5’-AAGATCACCAAGCCAACAGG-3’, reverse, 5’-TCTTTGGGGAGTTGAGGATG-3’. The fragments were 159 and 200 bp for *Gabra4* WT and *gabra4* CKO allele, respectively. The Institutional Animal Care and Use Committee, Chungnam National University School of Medicine, Daejeon, South Korea approved all procedures that involved animals (202109A-CNU-180, CNUH-021-A0011).

### Cells

Primary BMDMs, PMs, and Phoenix AMPHO (ATCC, CRL-3213) cells were cultured in Dulbecco’s modified Eagle’s medium (DMEM; Lonza; 12-604F) containing 10% fetal bovine serum (FBS; Gibco; 16000-044) and penicillin-streptomycin-amphotericin B (Lonza, 17-745E). Bone marrow cells harvested from each mouse were cultured in DMEM plus macrophage colony-stimulating factor (25 ng/mL, R&D Systems; 416-ML) for 6–7 days for BMDMs differentiation. Mouse PMs were cultured following a previously reported method [67]. Briefly, 1 mL of 3% thioglycollate was injected (i.p.) into each mouse for 3 days. The mice were euthanized and ice-cold PBS containing 3% FBS (5 mL) was injected into the peritoneal cavity; lavage was collected and centrifuged (1500 rpm, 8 min, 4°C) to obtain the macrophage pellet. AMs were isolated and cultured from bronchoalveolar lavage fluid. Briefly, 1 mL of PBS containing 3% EDTA was injected intratracheally to each euthanized mouse. Lavage was collected and centrifuged (1,500 rpm, 8 min, 4°C) to obtain the macrophage pellet. Cells were suspended in culture medium, counted, and cultured overnight.

### Bacterial strains and culture conditions

Mtb H37Rv was kindly provided by Dr. R. L. Friedman of the University of Arizona, *M. bovis* BCG was from the Korean Institute of Tuberculosis, and the smooth variant of Mabc was from ATCC (19977). They were grown in Middlebrook 7H9 broth (Difco, 271310) containing 0.5% glycerol, 0.005% Tween-80, and oleic albumin dextrose catalase (OADC; BD Biosciences, 212240). To culture Mtb strains encoding enhanced red fluorescent protein (ERFP) (Mtb-ERFP), Middlebrook 7H9 medium containing OADC and 50 μg/mL kanamycin (Sigma-Aldrich, 60615) was used. Bacterial suspensions were kept at −80°C in aliquots. In all experiments, mid-logarithmic-phase bacteria (absorbance 0.4) were used.

### Mycobacterial infection of macrophages and mice

Fully differentiated BMDMs, PMs, or AMs were infected with the indicated MOI of Mtb or Mabc for 2 h. To remove the extracellular bacteria, cells were washed using PBS and then further cultured for the indicated times in DMEM. For mycobacterial infection *in* *vivo*, bacteria were inoculated intranasally (Mtb; 5 × 10^4^ CFU/mice, BCG; 1 × 10^7^, Mabc; 1 × 10^7^ CFU/mice) to anesthetized mice. For neutrophil-neutralization experiments, mice were treated intraperitoneally with isotype control (clone 2A3, 0.2 mg) or a neutrophil-specific anti-Ly6G antibody (clone 1A8, 0.2 mg) at 24 h intervals 3 and 6 days post-infection with Mabc.

### CFU assays from macrophages and lungs

Using distilled water, cells were lysed to release intracellular bacteria, which were serially diluted and plated on Middlebrook 7H10 agar. For the evaluation of lung bacterial burden, mice were euthanized at 7 or 10 dpi for Mtb infection and 7 dpi for BCG and Mabc infection. The lungs were harvested, homogenized in PBS, serially diluted in PBS, and plated on 7H10 agar. Colonies formed in the plates were counted after 2–3 weeks of incubation (Mtb and BCG) and 3–5 days (Mabc).

### Sepsis mouse model

The murine sepsis model was established by i.p. injection of LPS (15 or 20 mg/kg). Mice were observed every 12 h for survival, and the overall survival rate was calculated. Mice were sacrificed after 6 h of LPS injection (14 mg/kg, i.p.) to harvest organs. Lung, liver, and spleen tissues were homogenized and used for RNA preparation.

### Depletion of macrophages

For macrophage depletion, clodronate-liposomes were used as described previously [3]. Briefly, clodronate-liposomes (200 μL i.p. and 25 μL i.n.) were injected to anesthetized *Gabra4* WT or *gabra4* CKO mice 24 h before infecting with Mabc (1 × 10^7^ CFU/mouse) to determine the CFU count in the lung (7 dpi).

### Reagents and chemicals

Anti-LAMP1 (sc-19992) and anti-ACTB (sc-47778) were bought from Santa Cruz Biotechnology. Anti-LC3A/B (PM036) was from MBL international. Anti-phospho-NF-κB (p65) (3033), anti-phospho-p44/42 MAPK (ERK1/2, 9101), anti-phospho-p38 (4511), anti-phospho-SAPK/JNK (4668), and anti-phospho-AMPKα Thr172 (2535) were bought from Cell Signaling. LPS (tlrl-eblps) and MSU (tlrl-msu) were obtained from InvivoGen. GABA (A2129), ATP (A5394), MitoTEMPO (SML0737), nigericin (SML-1779), isoniazid (I3377), bafilomycin A1 (B1793), and THIP (T101) were purchased from Sigma-Aldrich. 4′-6-Diamidino-2-phenylindole dihydrochloride (DAPI; D9542) and Alexa Fluor 488-conjugated anti-rabbit IgG (A17041), Alexa Fluor 594-conjugated anti-goat IgG (A11058), Fluo-4/AM (F14201), and MitoSOX Red (M36008) were obtained from Invitrogen. BAPTA-AM (196419) was from Calbiochem. Neutrophil antibodies (1A8; BP0075-1) and isotype control antibodies (2A3; BE0085) were purchased from Bio X Cell. Clodronate-liposomes (CLD-8909) were purchased from Encapsula Nano Sciences.

### Histology and immunohistochemistry

Lung tissues from *Gabra4* WT or *gabra4* CKO mice were harvested and fixed in 10% formalin. Then after embedding in paraffin wax, 4 μm thick sections were cut to use for hematoxylin and eosin staining, and images were captured using light microscopy. Sections were immunostained using an anti-mouse Ly6G antibody (Bio X Cell, Lebanon, NH; BP0075-1) to be used for confocal microscopy. Using FIJI software, the integrated intensity of the Ly6G-stained area in random fields was measured.

### Measurement of lactate

Supernatants from Mtb- or Mabc-infected (MOI 3) *Gabra4* WT or *gabra4* CKO PMs were collected, passed through 10 kDa cutoff filters (Millipore, UFC5010), and the extracellular level of lactate was determined with the Lactate Colorimetric/Fluorometric Assay Kit (BioVision, K607-100) following the protocol provided by the manufacturer.

### RNA preparation and real-time PCR

Total RNA preparation and cDNA synthesis was done using TRIzol reagent (Invitrogen, 15596026) and Superscript II Reverse Transcriptase (Invitrogen, 18064) respectively, following protocol provided by the manufacturer. Real-time PCR was conducted with SYBR Green Master Mix (Qiagen, 204074) in the Rotor-Gene Q 2plex system (Qiagen). Results are expressed as relative fold changes after analyzing by the 2^ΔΔ^ threshold cycle (Ct) method with *Gapdh* as an internal control. A list of the primer sequences can be found in Table S1.

### Western blotting

After washing with cold PBS, cells were lysed in RIPA buffer containing protease inhibitor cocktail (Roche, 11836153001) and phosphatase inhibitor cocktail (Sigma-Aldrich, P5726), and added with SDS sample buffer before boiling for 7 min. Proteins were subjected to SDS-PAGE before transferring to a PVDF membrane (Millipore, IPVH0001). Using 5% skim milk prepared in Tris-buffered saline - 0.1% Tween 20 (TBS-T), the membranes were blocked for 1 h at room temperature. After blocking, membranes were probed with primary antibodies at 4 °C overnight, and with appropriate secondary antibodies for 1 h at room temperature. Chemiluminescent signals were developed in a UVitec Alliance mini-chemiluminescence device (UVitec, UK) using Immobilon Western Chemiluminescent HRP Substrate (Millipore, WBKLS0500).

### ELISA

Cell supernatant was collected, centrifuged to discard debris, and stored at −80°C. Experiments were conducted following the manufacturers’ protocols using OptEIA Set ELISA Kit for IL-6 (555240; BD Biosciences) and the IL-1β ELISA kit (88-7013-88; Invitrogen).

### Extracellular flux analysis

The mitochondrial and glycolytic functions of macrophages were analyzed using a Seahorse Bioscience XF24 analyzer (Agilent Technologies), which evaluates the OCR and ECAR. PMs (2.5 × 10^5^ cells/well) were incubated overnight at 37°C before infecting them with Mabc (MOI 3) for 18 h. Next, the plate was incubated in a non-CO_2_ incubator for 1 h at 37°C after adding assay medium (590 µL/well). For ECAR analysis, XF base medium containing 1 mM L-glutamine, pH 7.4 was used, whereas, for OCR analysis, XF base medium with 2 mM L-glutamine, 1 mM sodium pyruvate, pH 7.4 was used. Activation of XF24 biosensor cartridge was done using XF24 calibrant solution (1 mL/well; Agilent Technologies) in a non-CO_2_ incubator for 24 h at 37°C. For OCR, after measuring basal OCR, 20 µg/mL oligomycin (ATPase inhibitor, final concentration 2 µg/mL), 50 µM CCCP (uncoupler, final concentration 5 µM), and 20 µM rotenone (mitochondrial complex I inhibitor, final concentration 2 µM) were injected serially to every well. For ECAR analysis, the basal ECAR was measured in a glycolysis stress test medium. Next, sequential injections of 100 mM glucose (to fuel glycolysis, final concentration 10 mM), 100 μM oligomycin (to inhibit ATP synthase in mitochondria, resulting in increased dependence on glycolysis, final concentration 10 μM), and 500 mM 2-deoxyglucose (2-DG, a competitive inhibitor of glucose, which suppresses glycolysis, final concentration 50 mM) were performed. OCR and ECAR analyses were completed at 37 °C. The data are presented per total number of cells.

### Generation of retrovirus expressing tandem LC3 plasmid

To measure autophagic flux, a tandem LC3B retroviral vector (mCherry-EGFP-LC3B) was produced as reported previously [69]. Using Lipofectamine 2000, phoenix AMPHO cells were co-transfected with the packaging plasmid pCL-Eco (0.75 μg; Addgene, 12371), envelope plasmid pMDG (0.25 μg; Addgene, 12259), and pBABE-puro mCherry-EGFP-LC3B plasmid (1 μg; Addgene, 22418) in a six-well plate. The medium was exchanged after 6 h. Retrovirus-containing cell supernatant was passed through a 0.45 μm syringe filter at 24 and 48 h after transfection.

### Immunofluorescence analysis and confocal microscopy

For autophagy analysis using immunofluorescence, cells were washed with PBS (3 times), fixed for 15 min, and permeabilized for 10 min, in 4% paraformaldehyde and 0.25% Triton X-100 (Sigma-Aldrich, T8787), respectively. Next, the samples were incubated with primary antibodies for 2 h at room temperature. Excess primary antibodies were removed with PBS and incubated for 1 h in secondary antibodies at room temperature. After mounting with Fluoromount-G mounting medium containing DAPI, fluorescence images were obtained in a confocal laser-scanning microscope (TCS SP8; Leica).

### Measurement of mtROS and intracellular Ca^2+^

Mtb-infected BMDMs were treated for 30 min with MitoSOX to measure mtROS. After washing with PBS, cells were mounted to a glass slide using a mounting medium containing DAPI. Images were visualized using a confocal laser-scanning microscope. To observe intracellular Ca^2+^, cells were incubated with the Ca^2+^ indicator Fluo-4/AM for 30 min in a 37°C incubator and infected with Mtb for 30 min or stimulated with LPS for 15 min. The intensity of Fluo-4/AM was measured by confocal microscopy.

### Image analysis

The phagosomal maturation was quantified after obtaining the micrographs using confocal laser-scanning microscopy and accompanied Leica software (LAS X; Leica). Non-co-localized in red and co-localized in yellow structures were counted to quantify the colocalization of mycobacteria with autophagosomes and lysosomes. Individual experiments involved duplicate coverslips. Quantitative results are presented as means with standard error of the mean (SEM). Image processing was performed using LAS X Small 2.0 and Adobe Photoshop 7 (Adobe Systems). Imaging parameters such as excitation, emission, pinhole, and exposure time were identical for all confocal laser scanning micrographs. The percentage of lysosomes, colocalization, and LC3 intensity were analyzed using ImageJ software (NIH).

### Transmission electron microscopy

After appropriate infection or stimulation, cells were washed with PBS before fixing for 1 h using 4% paraformaldehyde and 2% glutaraldehyde in 0.1 M sodium cacodylate buffer (pH 7.4). Post-fixation was performed in 1% osmium tetroxide and 0.5% potassium ferricyanide in cacodylate buffer for 1 h. Next, after embedding in resin, samples were cured for 24 h at 80°C. Using an ultramicrotome (RMC MT6000-XL), ultrathin sections (70–80 nm) were cut. The section staining was done with uranyl acetate and lead citrate, and then examined using a Bio-HVEM system (JEM-1400 Plus and JEM-1000 BEF; JEOL Ltd., Tokyo, Japan).

### Metabolome study

After appropriate infection, macrophages from *Gabra4* WT and *gabra4* CKO mice were washed with PBS (3 times). Next, 400 μL of methanol was added to each sample. Extraction of metabolite and subsequent metabolome analysis was performed at MetaMass (Seoul, Korea). Briefly, the internal standard (2-chlorophenylalanine, 1 mg/mL) was added to each sample, and the mixture was vortexed for 1 min. Samples were sonicated for 10 min and mixed for 10 min with a frequency set at 30 Hz in an MM400 Mixer Lill (Retsch®, Haan, Germany). For the next 1 h, the extracts were stored at 4°C and centrifuged for 10 min at 4°C with a speed of 13,000 rpm. After passing through a 0.2 μm pore polytetrafluoroethylene filter, supernatant was concentrated using a Modulspin 31 speed vacuum concentrator (Biotron, Seoul, South Korea). The dried samples were redissolved using methanol and 100 μL of extracts were re-dried in a speed vacuum concentrator (Biotron). Next, for oximation, 50 μL of methoxyamine hydrochloride (20 mg/mL in pyridine) was added and incubated for 90 min at 30°C. Thereafter, silylation of the reaction mixture was carried out by adding and incubating with N-methyl-N-(trimethylsilyl) trifluoroacetamide (MSTFA) (50 μL) for 30 min at 37°C, and by passing through a Millex GP 0.22 μm filter (Merck Millipore). GC-TOF/MS analysis was conducted using an Agilent 7890A system (Agilent Technologies) with an L-PAL3 autosampler and Pegasus III TOF-MS (Leco Corporation). The raw data of GC-TOF/MS analysis were obtained using LECO Chroma TOFTM software (version 4.44, LECO Corp.) and were converted into NetCDF format (*.cdf). Next, data were quantified and processed for peak selection, alignment, and baseline correction using Metalign software (http://www.metalign.nl). SIMCA-P+ (version 12.0; Umetrics) was utilized for multivariate statistical analyses. Comparison of metabolites was done with partial least squares-discriminant analysis (PLS-DA) modeling. Depending on variable importance in projection (VIP) scores > 1.0 and significance at *p* < 0.05, significantly discriminant variables were selected. Glycolysis-related metabolites (succinic acid, glyceric acid, methylsuccinic acid, and citric acid) were not identified because of the low VIP scores based on the KEGG database [70].

### Inflammasome analysis

PMs from *Gabra4* WT and *gabra4* CKO mice seeded in 24-well plates were sensitized using LPS (100 ng/mL) in Opti-MEM for 4 h before stimulating with ATP (5 mM), nigericin (10 μM), and MSU (200 μg/mL) for the indicated times. After collecting the supernatant, debris was removed by centrifuging, and stored at −80°C for ELISA or processed further for protein precipitation. Cell lysate was prepared in RIPA buffer for western blotting. Protein was precipitated from supernatant using StrataClean Resin (Agilent; 400724). Five hundred microliters of supernatant were taken from each sample and 5 μl of StrataClean Resin were added, mixed well by vortexing for 1 min, and centrifuged at 210 × g for 2 min at 4°C. The pellet was resuspended in 1× SDS sample buffer, boiled for 7 min, and subjected to western blotting.

### Flow cytometry

To analyze phagocytosis, BMDMs and PMs were infected with Mtb-ERFP for 4 h. After washing with PBS, cells were immediately subjected to flow cytometry using a FACS Canto II instrument (Becton Dickinson), and the data analysis was done using FlowJo software (Tree Star).

### Lentiviral sh*Ampk* production and viral transduction

AMPKα was knocked down in mouse primary cells using an shRNA plasmid (Santa Cruz Biotechnology, sc-29674-SH). The lentiviral vector was produced by transfecting 293T cells using Lipofectamine 3000 (L3000008; Invitrogen) in the presence of RSV-Rev, pMD2.G, pMDLg/pRRE. After 6 h of incubation in 5% CO_2_ at 37°C, the medium was replaced. After 48 h, supernatant containing the virus was collected, centrifuged at 2,000 for 10 min, and passed through 45 μM filters to eliminate cell debris. They were then aliquoted and stored at −80°C. Transduction efficiency was analyzed by qRT-PCR.

## Statistical analysis

All the data analysis was performed in GraphPad Prism (version 5.0, 8.4.0, or 8.4.3). Significant differences were evaluated by two-tailed Student’s *t* test, ANOVA, or Mann-Whitney U test. For *in vivo* survival data, the log-rank (Mantel-Cox) test was performed to evaluate significance. The significance of differences in PLS-DA data was evaluated by analysis of variance using PASW Statistics 18 software (SPSS Inc.). Bar plots were created based on the relative peak areas of distinct metabolite masses by STATISTICA 7 software (StatSoft Inc) and one-way ANOVA with Tukey’s multiple comparison test in GraphPad Prism. All reported results were replicable.

## Supplementary Material

Supplemental Material
