# Supplementary material for: Gamma-aminobutyric acid type A receptor alpha 4 coordinates autophagy, inflammation, and immunometabolism to promote innate immune activation
Source: Autophagy Rep. 2023 Mar 1;2(1):2181915. doi: 10.1080/27694127.2023.2181915 (PMC12042478; doi:10.1080/27694127.2023.2181915)
Supplement: Supplemental Material [file KAUO_A_2181915_SM2274.docx]

**Supplemental file for:**

**Gamma-aminobutyric acid type A receptor alpha 4 coordinates autophagy, inflammation, and immunometabolism to promote innate immune activation** Running Title: *Gabra4* regulation of innate immune response

**Supplementary figures and legends**

**
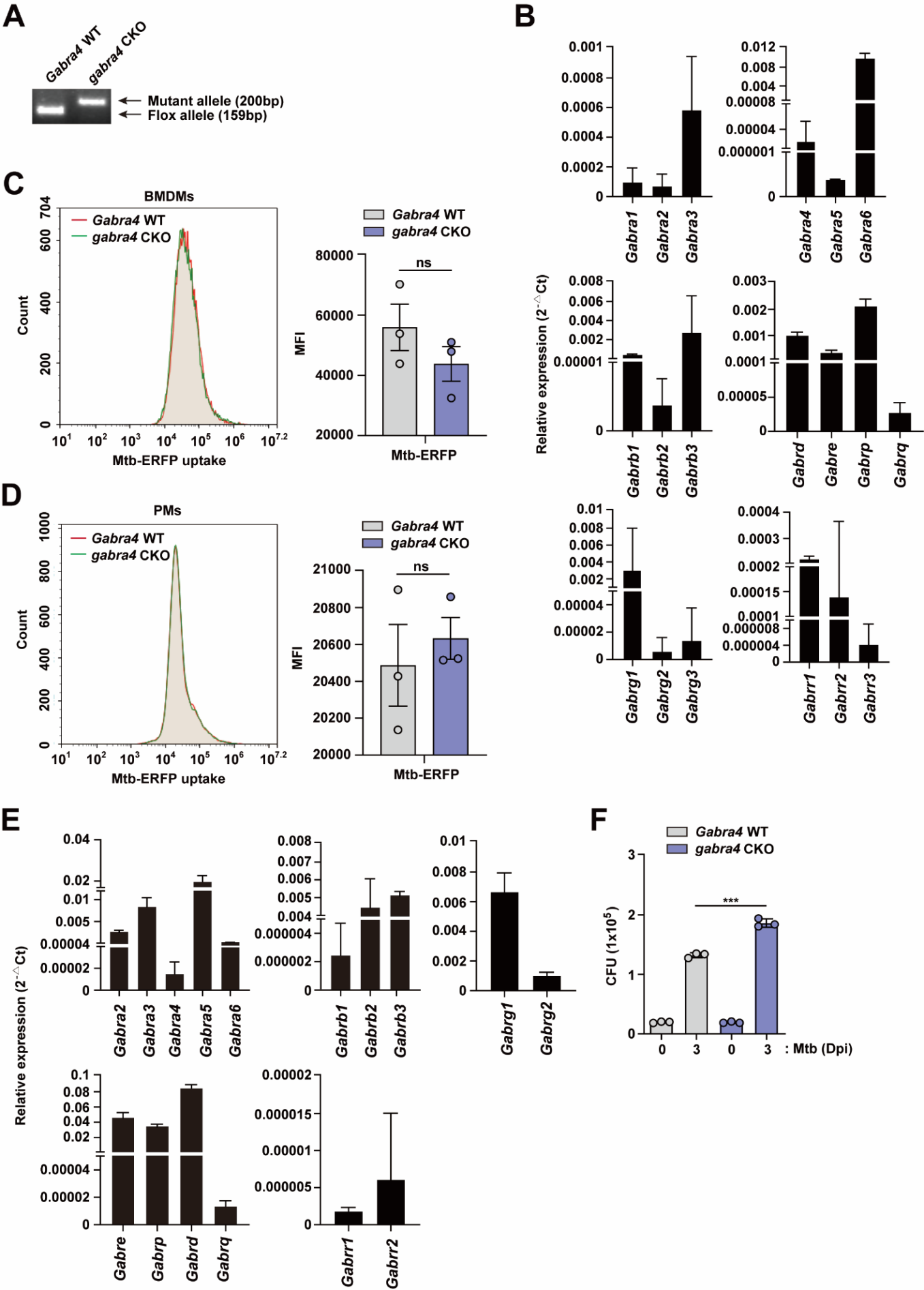
**

**Figure S1**. Confirmation of genotying, phagocytosis analysis, gene expression profiling of GABA_A_R subunits, and intracellular survival of Mtb in *Gabra4* WT and *gabra4* CKO macrophages. (**A**) PCR analysis of genomic DNA from *Gabra4* WT and *gabra4* CKO mice. (**B**) Relative expression (2 delta Ct) values of several GABA_A_R subunits in PMs. (**C, D**) Phagocytosis measured by flow cytometry in *Gabra4* WT and *gabra4* CKO BMDMs (C) or PMs (D) infected with Mtb-ERFP for 4 h. (**E**) Relative expression (2 delta Ct) values of several GABA_A_R subunits in AMs. (**F**) Intracellular bacterial growth in AMs from *Gabra4* WT or *gabra4* CKO mice after Mtb (MOI 1) infection. Mean ± SEM (C-D) and ± SD (F) are shown. The two-tailed Student’s *t* tests (C-D) and One-way ANOVA (F) were used to measure the significance. Data shown are the representative of three independent experiments. ****p* < 0.001. Dpi, days post infection; MFI, mean fluorescence intensity; ns, not significant.


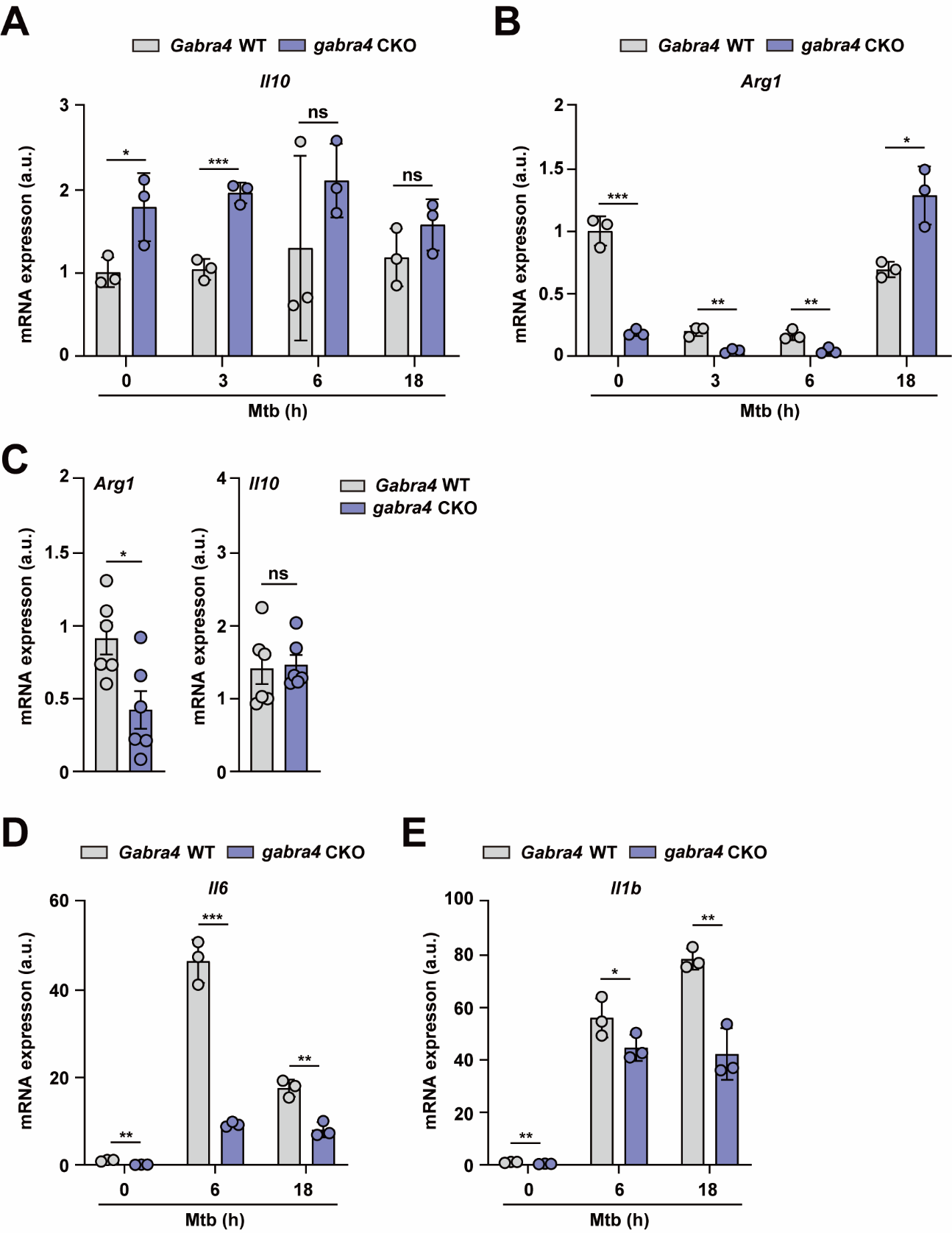


**Figure S2.** GABRA4-mediated regulation of inflammatory responses in macrophages and in the lungs. (**A, B**) qRT-PCR analysis of *Il10* and *Arg1* mRNA expression in *Gabra4* WT and *gabra4* CKO BMDMs after Mtb (MOI 1) infection for the indicated time. (**C**) mRNA expression of *Arg1* and *Il10* in the lungs of *Gabra4* WT and *gabra4* CKO mice infected with Mtb (5 × 10^4^ CFU) at 10 dpi. (**D, E**) qRT-PCR analysis of *Il6* and *Il1b* mRNA expression in *Gabra4* WT and *gabra4* CKO AMs after Mtb (MOI 1) infection for the indicated time. The Students *t*-test (A, B, D and E) or Mann-Whitney U test (C) were used to measure the significance. Data shown are the representative of three independent experiments. **p* < 0.05, ***p* < 0.01, ****p* < 0.001. ns, not significant.


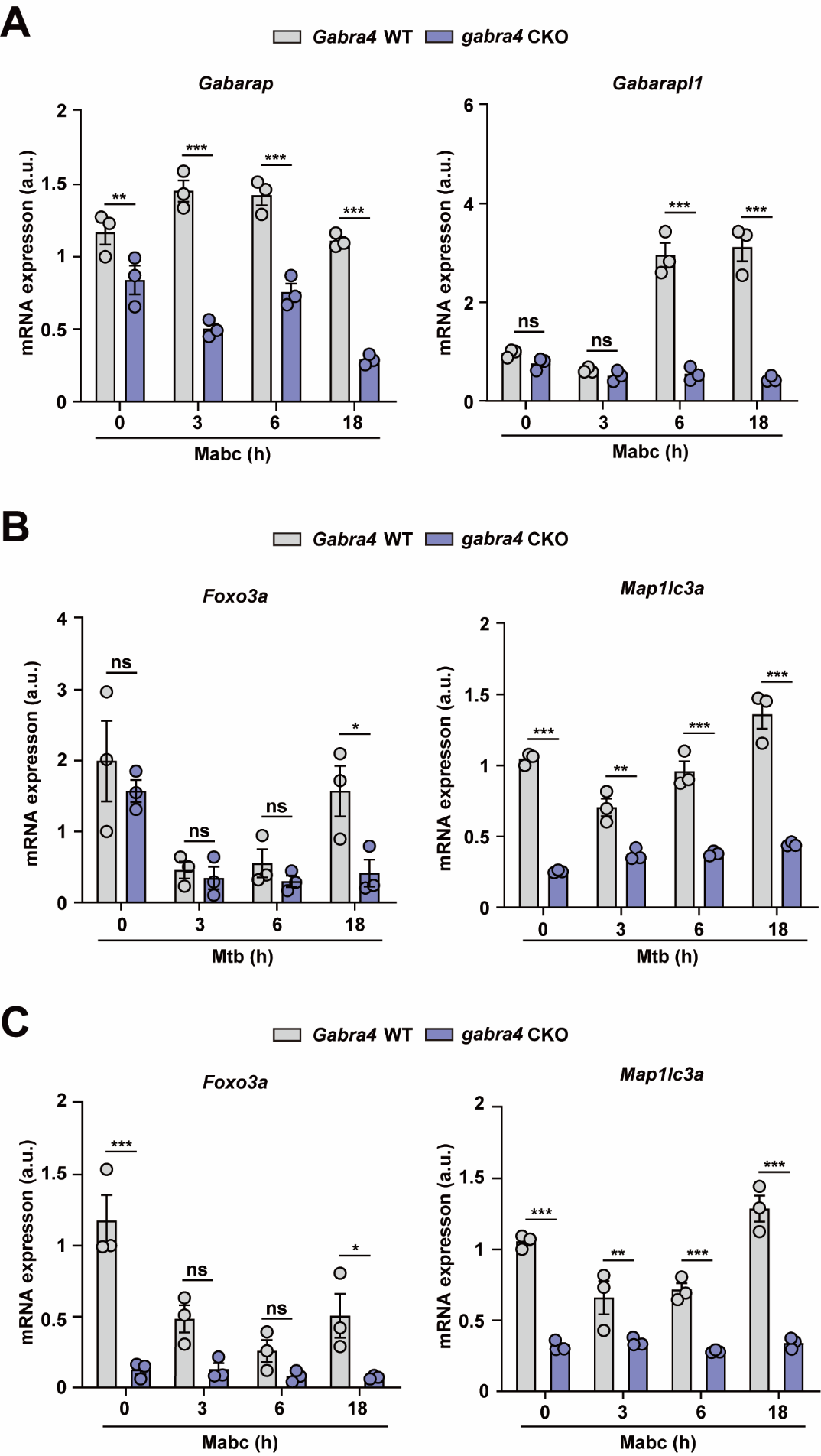


**Figure S3**. GABRA4 is essential for the expression of autophagy-related genes in macrophages during mycobacterial infection. (**A**) qRT-PCR analysis of *Gabarap* and *Gabarapl1* in *Gabra4* WT and *gabra4* CKO PMs after Mabc (MOI 3) infection for the indicated time. (**B**) qRT-PCR analysis of *Foxo3a* and *Map1lc3a* in *Gabra4* WT and *gabra4* CKO PMs after Mtb (MOI 1) infection for the indicated time. (**C**) qPCR analysis of *Foxo3a* and *Map1lc3a* in *Gabra4* WT and *gabra4* CKO PMs after Mabc (MOI 3) infection for the indicated time. Mean ± SEM are shown. Two-way ANOVA was used to measure the significance (A-C). Data shown are the representative of three independent experiments. **p* < 0.05, ***p* < 0.01, ****p* < 0.001. ns, not significant.


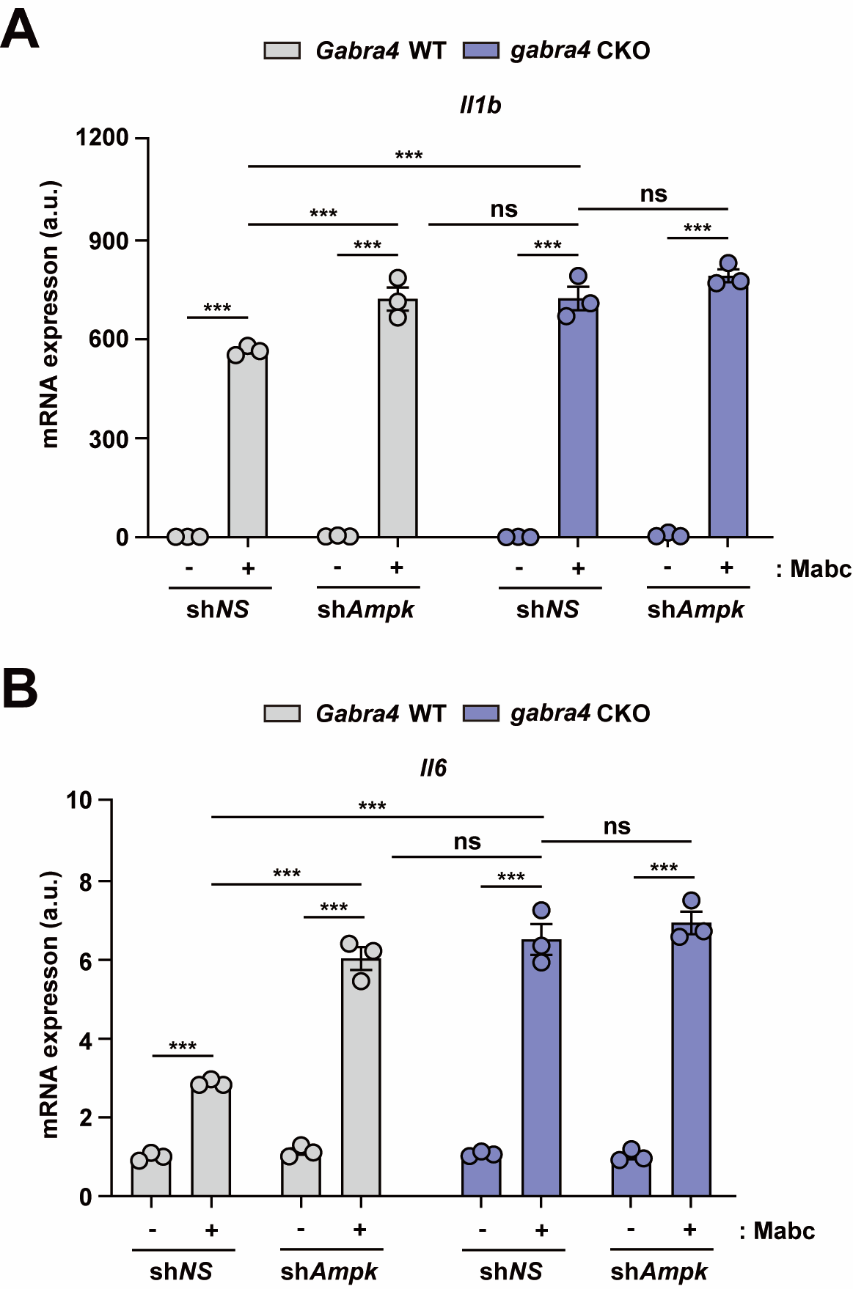


**Figure S4.** GABRA4-mediated AMPK signaling contributes to controlling inflammatory responses in macrophages during mycobacterial infection. (**A, B**) *Gabra4* WT or *gabra4* CKO macrophages transduced with sh*NS* or sh*Ampk* and infected with Mabc (MOI 3) for 18 h to measure the mRNA expression of *1l1b* (A) and *Il6* (B). Data are means ± SD. One-way ANOVA was used to assess significance. Data shown are the representative of three independent experiments. ****p* < 0.001. ns, not significant.


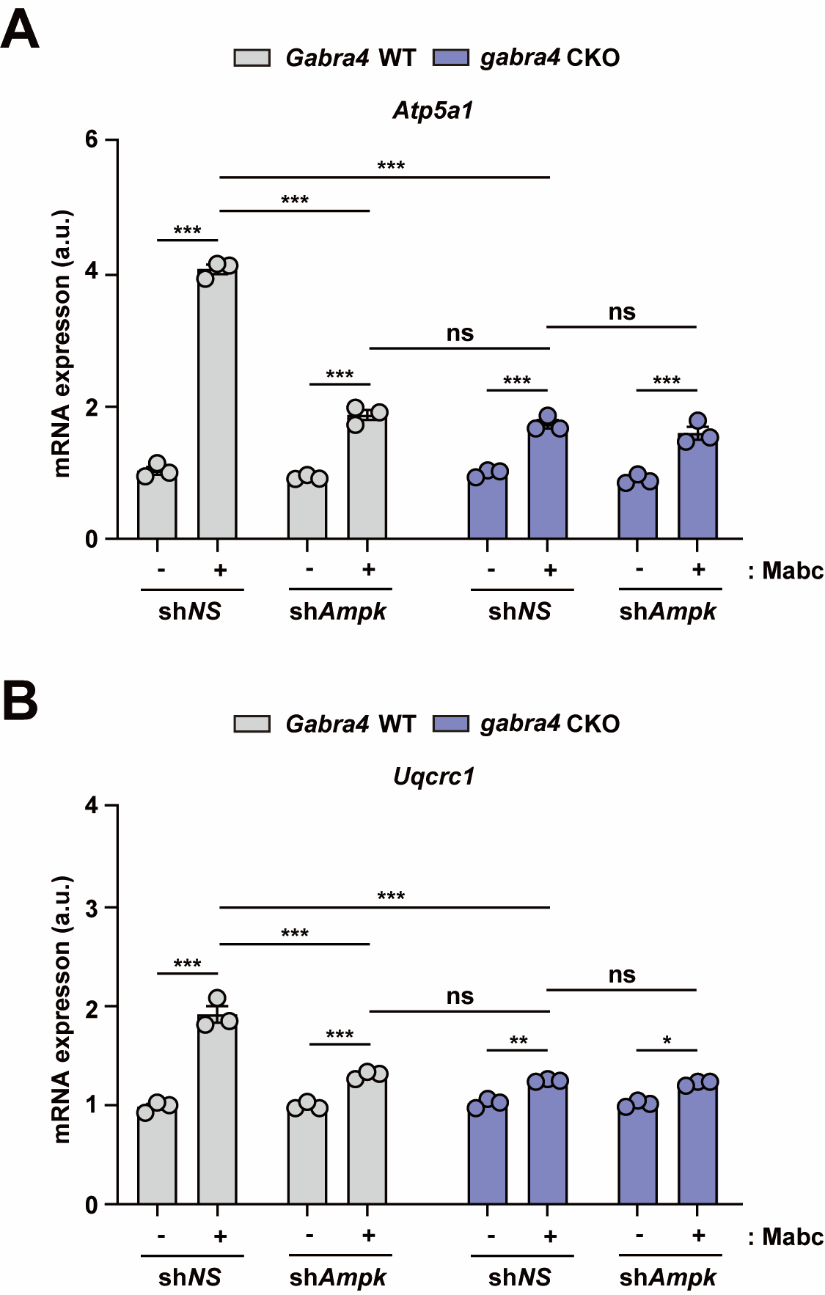


**Figure S5.** GABRA4-mediated AMPK signaling contributes to regulate *Uqcrc1* and *Atp5a1* expression in macrophages during mycobacterial infection. (**A, B**) *Gabra4* WT or *gabra4* CKO macrophages transduced with sh*NS* or sh*Ampk* and infected with Mabc (MOI 3) for 18 h to measure the mRNA expression of *Atp5a1* (A) and *Uqcrc1* (B). Data are means ± SD. One-way ANOVA was used to assess significance. Data shown are the representative of three independent experiments. **p* < 0.05, ***p* < 0.01, ****p* < 0.001. ns, not significant.


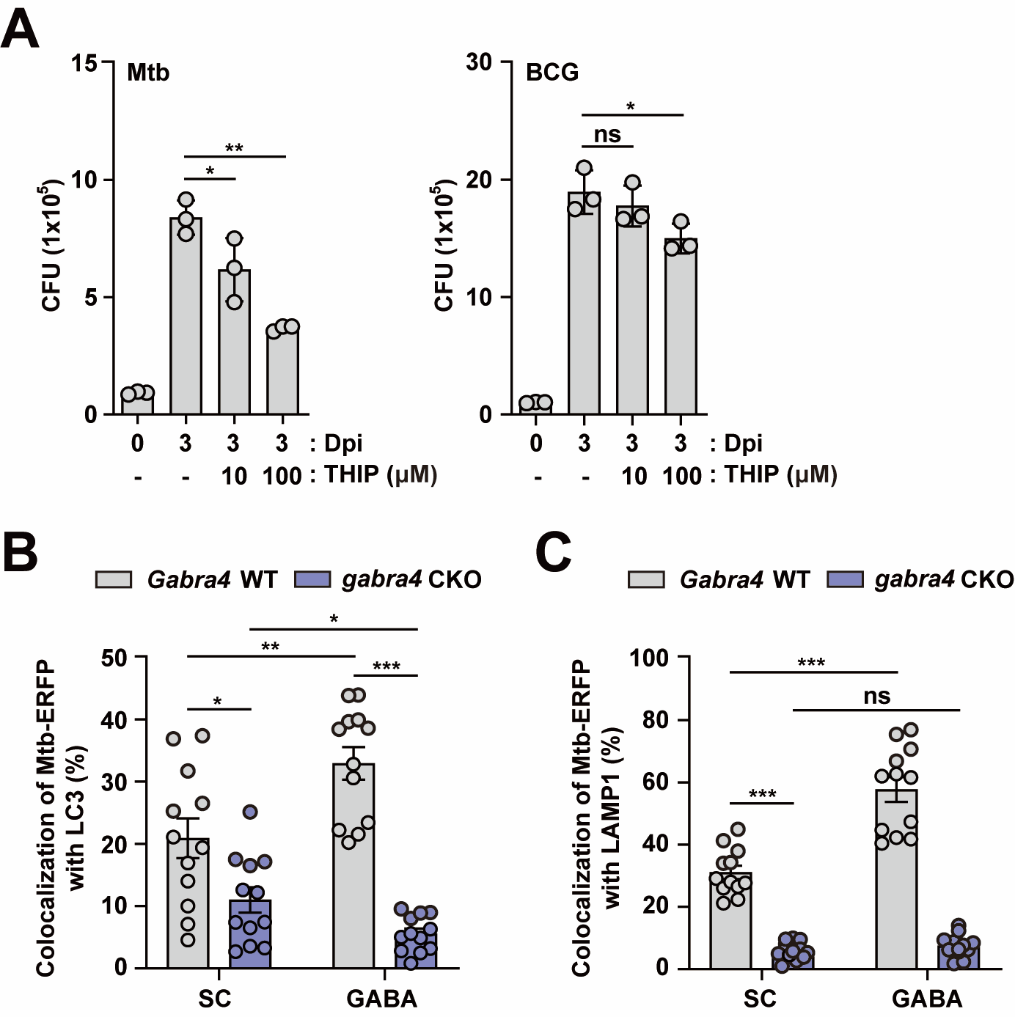


**Figure S6**. The pharmacological activation of GABA_A_R promotes antimicrobial responses and xenophagy activation in macrophages depending on the GABRA4. (**A**) Intracellular survival assay in PMs-infected with Mtb (MOI 1) or BCG (MOI 1) in the presence of THIP (10 and 100 μM) for the indicated time. (**B, C**) PMs from *Gabra4* WT and *gabra4* CKO mice were infected with Mtb-ERFP (MOI 5), incubated with GABA (100 μM) for 18 h, then stained with LC3 (B) or LAMP1 (C). (B) Quantitative data of colocalization of Mtb-ERFP with LC3. (C) Quantitative data of colocalization of Mtb-ERFP with LAMP1. Mean ± SEM is shown (A-C). One-way ANOVA (A) and two-way ANOVA (B, C) were used to measure the significance. Data shown are the representative of three independent experiments. **p* < 0.05, ***p* < 0.01, ****p* < 0.001. Dpi, days post infection; SC, solvent control; ns, not significant.


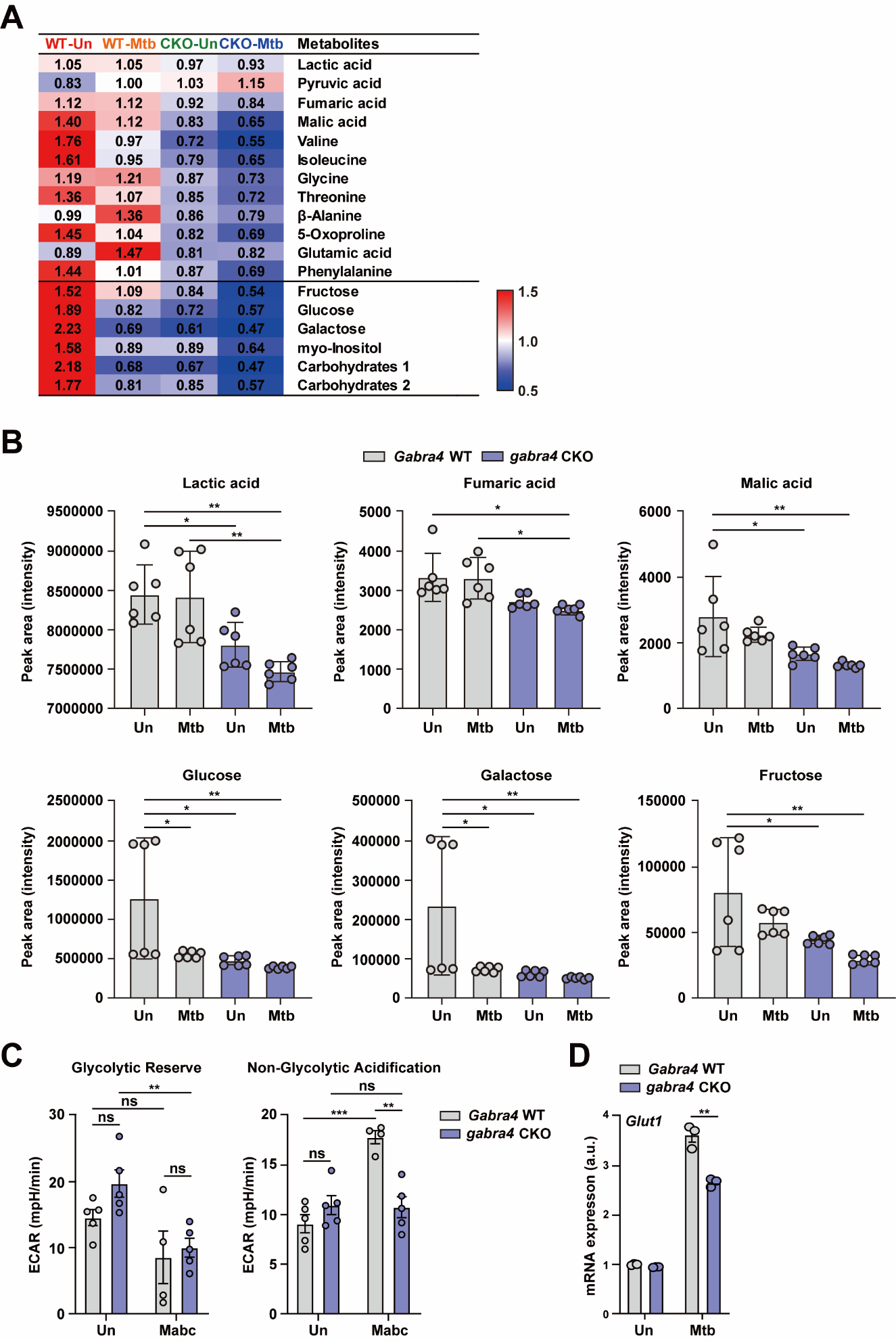


**Figure S7.** *Gabra4* deficiency results in the depletion of organic acids and sugar in macrophages during Mtb infection. (**A**) Heatmap of the discriminate metabolites among the experimental groups with the relative concentration of metabolites from *Gabra4* WT and *gabra4* CKO macrophages based on the PLS-DA model (VIP score > 1.0, *p* < 0.05). The values in the heatmap represent fold changes normalized to the average value of each metabolite. Metabolites significantly decreased were displayed in blue, while metabolites significantly increased were displayed in red. (**B**) The bar plots of organic acids and sugars showed relative abundance that was calculated using relative peak area from GC-TOF/MS. (**C**) ECAR parameters calculated from the Fig. 7E are shown. (**D**) mRNA expression of *Glut1* in the Mtb-infected PMs from *Gabra4* WT or *gabra4* CKO mice. The data were analyzed by the one-way ANOVA and Tukey’s multiple comparison test (A, B) or Student’s *t* test (C, D). Data shown are the representative of three independent experiments. **p* < 0.05, ***p* < 0.01, ****p* < 0.001. Un, untreated; ns, no significant.

**
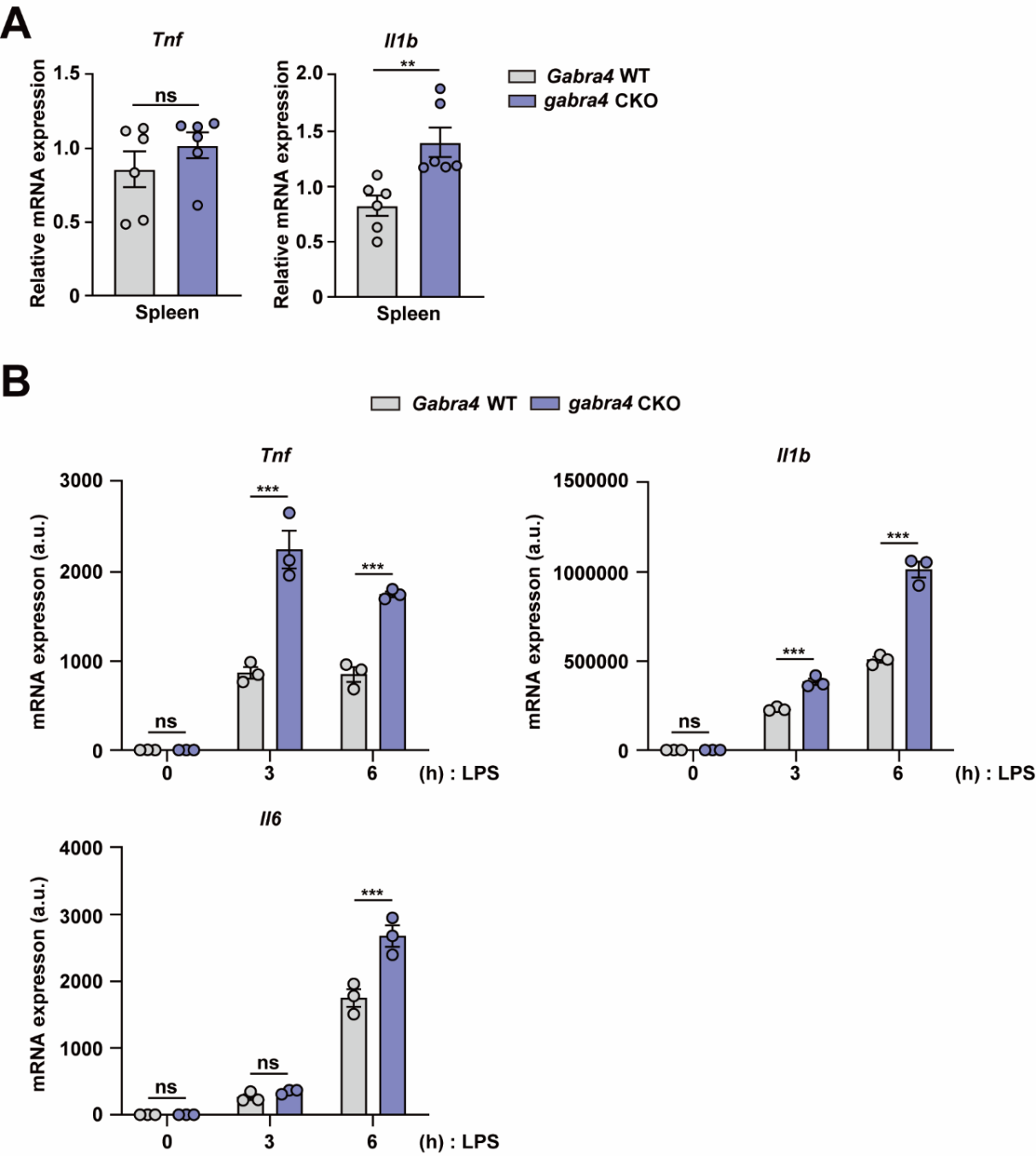
**

**Figure S8**. GABRA4 is essential for controlling inflammatory responses after LPS treatment in macrophages. (**A**) qRT-PCR analysis of *Tnf* and *Il1b* in spleen of *Gabra4* WT and *gabra4* CKO mice injected with LPS (14 mg/kg, i.p.) for 6 h. (**B**) qRT-PCR analysis of *Tnf*, *Il1b,* and *Il6* in *Gabra4* WT and *gabra4* CKO PMs stimulated with LPS (100 ng/ml) for the indicated time. Mean ± SEM is shown (A, B). Two-way ANOVA (A) and two-tailed Student’s *t* tests (B) were used to measure the significance. Data shown are the representative of three independent experiments. ***p* < 0.01 and ****p* < 0.001. ns, not significant.


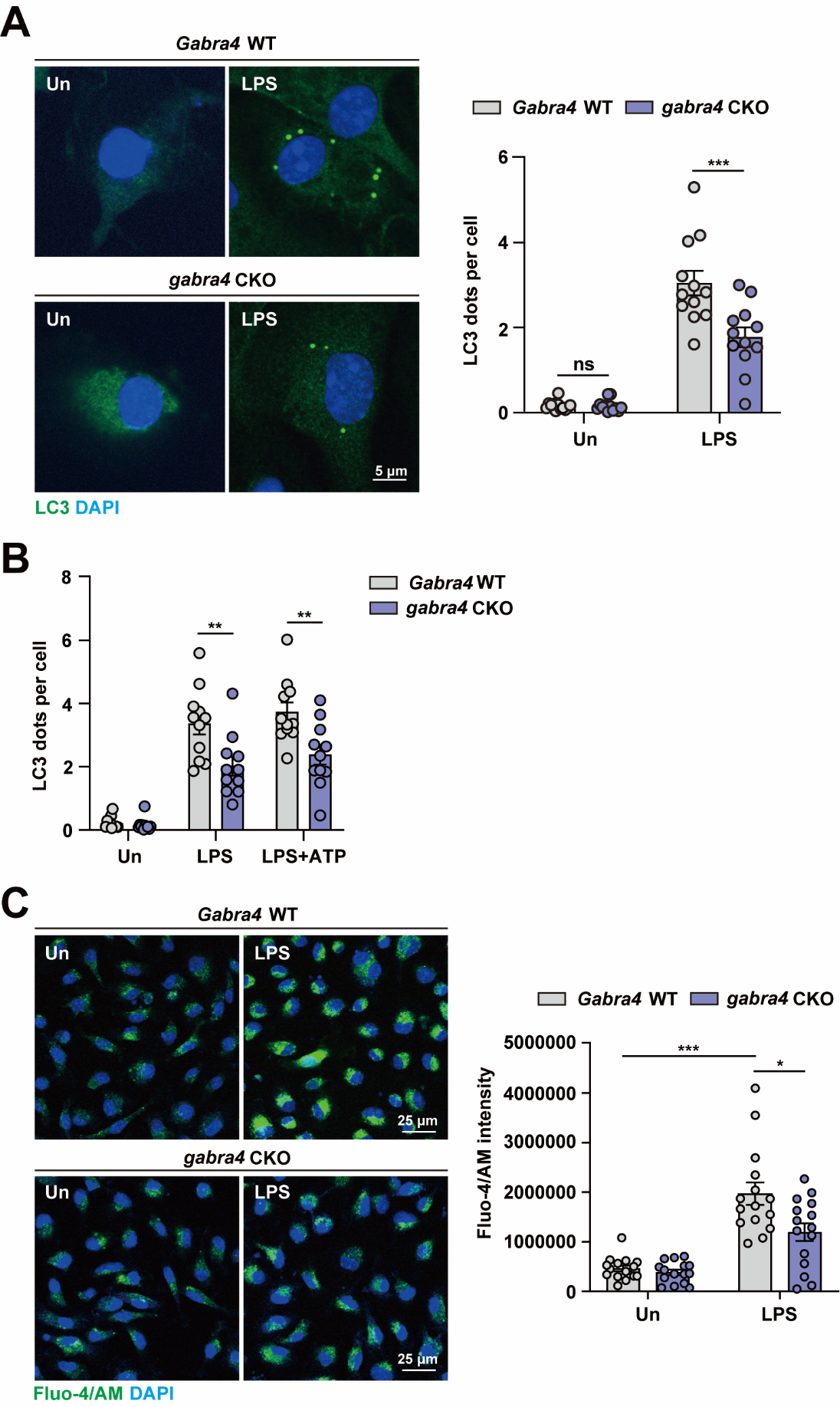


**Figure S9**. GABRA4 regulates LPS-induced autophagy and intracellular calcium release in macrophages. (**A**) PMs from *Gabra4* WT and *gabra4* CKO mice were incubated with LPS (100 ng/ml) for 6 h and cells were stained with LC3 and DAPI (for nuclei; blue). Cells were visualized by confocal microscopy. Scale bar, 5 μm (left). Quantitative data of LC3 dots per cell (right). (**B**) PMs from *Gabra4* WT and *gabra4* CKO mice were incubated with LPS (100 ng/ml) for 4 h and incubated without or with ATP (5 mM) for 30 min. Cells were stained with LC3 and analyzed by confocal microscopy. Quantitative data of LC3 dots per cell. (**C**) BMDMs from *Gabra4* WT and *gabra4* CKO mice were incubated with Fluo-4/AM and treated with LPS (100 ng/ml). Representative images. Scale bar, 25 μm (left). Quantitative data of Fluo-4/AM intensity (right). The two-tailed Student’s *t* tests (A-C) was used to measure the significance. Data shown are the representative of three independent experiments. **p* < 0.05, ***p* < 0.01, ****p* < 0.001. Un, untreated; ns, not significant.

**Table S1.** Primers used in this study.

| Genes | Primer | Sequences |
| --- | --- | --- |
| *Ampk* | Forward  Reverse | 5’-TGTTCCAGCAGATCCTTTCC-3’  5’-ATAATTGGGTGAGCCACAGC-3’ |
| *Arg1* | Forward  Reverse | 5’-CTCCAAGCCAAAGTCCTTAGAG-3’  5’-AGGAGCTGTCATTAGGGACATC-3’ |
| *Atp5a1* | Forward  Reverse | 5’-CATTGGTGATGGTATTGCGC-3’  5’-TCCCAAACACGACAACTCC-3’ |
| *Ccl2* | Forward  Reverse | 5’-TGACCCCAAGAAGGAATGGG-3’  5’-ACCTTAGGGCAGATGCAGTT-3’ |
| *Cxcl5* | Forward  Reverse | 5’-CCGCTGGCATTTCTGTTGCTGT-3’  5’-CAGGGATCACCTCCAAATTAGCG-3’ |
| *Foxo3a* | Forward  Reverse | 5’-GAGCTGGAGCTCGAACCTT-3’  5’-TCCACTTCGAGCGGAGAGAG-3’ |
| *Gabarap* | Forward  Reverse | 5’-AAGAGGAGCATCCGTTCGAGA-3’  5’-GCTTTGGGGGCTTTTTCCAC-3’ |
| *Gabarapl1* | Forward  Reverse | 5’-TGACCCCAAGAAGGAATGGG-3’  5’-ATACAGCTGGCCCATGGTAG-3’ |
| *Gabra1* | Forward  Reverse | 5’-AAAAGCGTGGTTCCAGAAAA-3’  5’-GCTGGTTGCTGTAGGAGCAT-3’ |
| *Gabra2* | Forward  Reverse | 5’-GCTACGCTTACACAACCTCAGA-3’  5’-GACTGGCCCAGCAAATCATACT-3’ |
| *Gabra3* | Forward  Reverse | 5’-GCCGTCTGTTATGCCTTTGTATTT-3’  5’-TTCTTCATCTCCAGGGCCTCT-3’ |
| *Gabra4* | Forward  Reverse | 5’-AGAACTCAAAGGACGAGAAATTGT-3’  5’-TTCACTTCTGTAACAGGACCCC-3’ |
| *Gabra5* | Forward  Reverse | 5’-GATTGTGTTCCCCATCTTGTTTGGC-3’  5’-TTACTTTGGAGAGGTGGCCCCTTTT-3’ |
| *Gabra6* | Forward  Reverse | 5’-GGTGACCGGGCATCCCAGTGA-3’  5’-TGTTACAGCACCCCCAAATCCTGGC-3’ |
| *Gabrb1* | Forward  Reverse | 5’-GGTTTGTTGTGCACACAGCTCC-3’  5’-CATGCGCACGGCGTACCAAA-3’ |
| *Gabrb2* | Forward  Reverse | 5’-GCTGGTGAGGAAATCTCGGTCCC-3’  5’-GACTGGCCCAGCAAATCATACT-3’ |
| *Gabrb3* | Forward  Reverse | 5’-GAGCGTAAACGACCCCGGGAA-3’  5’-GGGACCCCCGAAGTCGGGTCT-3’ |
| *Gabrg1* | Forward  Reverse | 5’-ATCCACTCTCATTCCCATGAACAGC-3’  5’-ACAGAAAAAGCTAGTACAGTCTTTGC-3’ |
| *Gabrg2* | Forward  Reverse | 5’-ACTTCTGGTGACTATGTGGTGAT-3’  5’-GGCAGGAACAGCATCCTTATTG-3’ |
| *Gabrg3* | Forward  Reverse | 5’-ATTACATCCAGATTCCACAAGATG-3’  5’-CACAGGTGTCCTCAAATTCCT-3’ |
| *Gabrd* | Forward  Reverse | 5’-TCAAATCGGCTGGCCAGTTCCC-3’  5’-GCACGGCTGCCTGGCTAATCC-3’ |
| *Gabre* | Forward  Reverse | 5’-ACTGCGCCCTGGCATTGGAG-3’  5’-AGGCCCGAGGCTGTTGACAA-3’ |
| *Gabrq* | Forward  Reverse | 5’-GCTGGAGGTGGAGAGCTATGGCT-3’  5’-CCCCAGGTACGTGTACTGAGGGA-3’ |
| *Gabrp* | Forward  Reverse | 5’-TCGGTGGTGACCCAGTTCGGAT-3’  5’-TCTGTCCAACGCTGCCGGAG-3’ |
| *Gabrr1* | Forward  Reverse | 5’-CCATCTAGGAAAGGCAGCAG-3’  5’-GAGCTTCGTCTCAGGATTGG-3’ |
| *Gabrr2* | Forward  Reverse | 5’-GCTGCCTGTTGCATCATAGA-3’  5’-ATACAAATGGCTTGGCTTGG-3’ |
| *Gabrr3* | Forward  Reverse | 5’-CAACTCAACAGGAGGGGAAA-3’  5’-TCCACATCAGTCTCGCTGTC-3’ |
| *Gapdh* | Forward  Reverse | 5’-CATCGTGGAGAAGGCTCCTA-3’  5’-TGGCAAAGTGGAGATTGTTGCC-3’ |
| *Hif1a* | Forward  Reverse | 5’-CAAGATCTCGGCGAAGCAA-3’  5’-GGTGAGCCTCATAACAGAAGCTTT-3’ |
| *Il1b* | Forward  Reverse | 5’-TACGGACCCCAAAAGATGA-3’  5’-TGCTGCTGCGAGATTTGAAG-3’ |
| *Il6* | Forward  Reverse | 5’-TACCACTTCACAAGTCGGAGGC-3’  5’-CTGCAAGTGCATCATCGTTGTTC-3’ |
| *Il10* | Forward  Reverse | 5’-GCTCTTGCACTACCAAAGCC-3’  5’-CTGCTGATCCTCATGCCAGT-3’ |
| *Ldha* | Forward  Reverse | 5’-TGCCTACGAGGTGATCAAGCT-3’  5’-GCACCCGCCTAAGGTTCTTC-3’ |
| *Map1lc3a* | Forward  Reverse | 5’-GACCGCTGTAAGGAGGTGC-3’  5’-CTTGACCAACTCGCTCATGT-3’ |
| *Tnf* | Forward  Reverse | 5’-ACGGCATGGATCTCAAAGAC-3’  5’-AGATAGCAAATCGGCTGACG-3’ |
| *Uqcrc1* | Forward  Reverse | 5’-ATCAAGGCACTGTCCAAGG-3’  5’-TCATTTTCCTGCATCTCCCG-3’ |
